# Mucin 4 Confers Gemcitabine Resistance and an Unfavorable Prognosis in Patients with Cholangiocarcinoma via AKT Activation

**DOI:** 10.7150/ijbs.79126

**Published:** 2023-05-21

**Authors:** Yi-Ru Pan, Chiao-En Wu, Shih-Ming Jung, Shih-Chiang Huang, Sheng-Hsuan Lin, Wen-Chi Chou, Yu-Chan Chang, Ming-Huang Chen, Tsai-Hsien Hung, Alice L. Yu, Wen-Kuan Huang, Chun-Nan Yeh

**Affiliations:** 1 Department of Surgery, Chang Gung Memorial Hospital, Linkou, Chang Gung University, Taoyuan 333, Taiwan.; 2 Division of Hematology-Oncology, Department of Internal Medicine, Chang Gung Memorial Hospital, Linkou, Chang Gung University College of Medicine, Taoyuan 333, Taiwan.; 3 Department of Pathology, Chang Gung Memorial Hospital, Linkou, Taoyuan 333, Taiwan.; 4 Department of Biomedical Imaging and Radiological Sciences, National Yang Ming Chiao Tung University, Taipei 112, Taiwan.; 5 Center for Immuno-Oncology, Department of Oncology, Taipei Veterans General Hospital, Taipei 112, Taiwan.; 6 School of Medicine, National Yang Ming Chiao Tung University, Taipei 112, Taiwan.; 7 Institute of Stem Cell and Translational Cancer Research, Chang Gung Memorial Hospital at Linkou, Chang Gung University, Taiwan.; 8 Department of Pediatrics, University of California in San Diego, San Diego, CA 92103, USA.; 9 School of Medicine, National Tsing Hua University, Hsinchu 30013, Taiwan.

**Keywords:** Mucin 4, plasma, cholangiocarcinoma, GEM resistance, AKT inhibitor

## Abstract

Cholangiocarcinoma (CCA) exhibits aggressive biological behavior and a poor prognosis. Gemcitabine (GEM)-based chemotherapy is the first-line chemotherapy for advanced CCA but has a response rate of only 20-30%. Therefore, investigating treatments to overcome GEM resistance in advanced CCA is crucial. Among mucin (MUC) family members, MUC4 showed the greatest increase in the resistant versus parental sublines. MUC4 was upregulated in whole-cell lysates and conditioned media from gemcitabine-resistant (GR) CCA sublines. MUC4 mediated GEM resistance by activating AKT signaling in GR CCA cells. The MUC4-AKT axis induced BAX S184 phosphorylation to inhibit apoptosis and downregulated GEM transporter human equilibrative nucleoside transporter 1 (hENT1) expression. The combination of AKT inhibitors and GEM or afatinib overcame GEM resistance in CCA. In vivo, capivasertib (an AKT inhibitor) increased GEM sensitivity in GR cells. MUC4 promoted EGFR and HER2 activation to mediate GEM resistance. Finally, MUC4 expression in patient plasma correlated with MUC4 expression. Paraffin-embedded specimens from non-responders expressed significantly more MUC4 than did those from responders, and this upregulation was associated with poor progression-free survival and overall survival. In GR CCA, high MUC4 expression promotes *sustained* EGFR/HER2 signaling and AKT activation. The combination of AKT inhibitors with GEM or afatinib might overcome GEM resistance.

## Introduction

Cholangiocarcinoma (CCA) is the most common biliary tract cancer (BTC). CCA is a malignant tumor from cholangiocytes in bile duct branches and is divided into three subtypes: intrahepatic CCA (iCCA), perihilar CCA (pCCA), and distal CCA (dCCA) 1-3. CCA is the second most common primary hepatic malignancy with aggressive biological behavior and has a relatively poor prognosis because it is diagnosed at an advanced stage 4, 5. Gemcitabine (GEM)-based chemotherapy is the standard of care for patients with advanced CCA 6-8, but primary or acquired resistance to GEM compromises therapeutic efficacy 9-11. Potential targets that have been identified based on tumor genomic profiling, including FGFR2 fusion, IDH1 mutation, NTRK fusion, and BRAF mutation, provide therapeutic options after intolerance to or failure of GEM-based chemotherapy 12. Previous studies have shown that the infiltration of immunosuppressive immune cells is associated with poor prognosis in CCA patients 13. Pembrolizumab, an anti-PD-1 antibody, is an FDA-approved immune checkpoint inhibitor (ICI) for various cancers and provides a limited response rate of 5.8% among advanced BTC patients (KEYNOTE-158) 14. In light of this limited efficacy, clinical trials to assess the combination of ICI with GEM-based chemotherapy have been conducted 15. The TOPAZ-1 and T1219 studies recently showed a positive result: ICI plus chemotherapy demonstrated longer survival than that achieved with traditional chemotherapy 16, 17. However, the combination of ICIs and chemotherapy was not appropriate for the patients with advanced BTCs and GEM resistance remains the main clinical challenge. Therefore, it is crucial to explore subsequent therapies for patients with advanced CCA refractory to GEM-based chemotherapy.

Mucins (MUCs), high molecular weight O-glycoproteins, are typically expressed at the apical surface of epithelial cells 18 and include three subfamilies: membrane-bound/transmembrane mucins, secreted (gel-forming) mucins, and soluble mucins 19. Among MUC proteins, MUC1, MUC2, MUC4, MUC5AC, MUC6, MUC13, and MUC16 have been demonstrated to be involved in cancer progression 20. Circulating N-terminal MUC1 (CA15-3) is used for monitoring the clinical course in breast cancer 21, and circulating MUC16 (CA125) is used for detecting early-stage disease and monitoring the clinical course in ovarian cancer 22. MUC4 has been identified as a potential marker for the diagnosis of pancreatic cancer and is associated with a poor prognosis 23. Moreover, the increased expression of MUC4 correlates with a poor outcome in patients with CCA and extrahepatic bile duct carcinoma 24-26. High MUC protein expression was found be associated with malignant transformation 27. Drug resistance can develop during cancer transformation 28. In pancreatic cancer, MUC1, MUC4, and MUC 5AC mediate GEM sensitivity 29-31. However, the role of mucin proteins in GEM resistance in CCA remains unclear. In this study, we demonstrated that GEM-resistant (GR) CCA with high MUC4 expression induced sustained EGFR/HER2 signaling, resulting in AKT activation. The combination of an AKT inhibitor with GEM or afatinib might overcome GEM resistance in patients with advanced CCA.

## Materials and Methods

### Cells

SSP-25 cells were purchased from the RIKEN Cell Bank (Ibaraki, Japan). SNU-1196 cells were purchased from the Korean Cell Line Bank (Seoul, Korea). The SSP-25 and SNU-1196 cell lines were grown in RPMI medium supplemented with 10% fetal bovine serum and penicillin-streptomycin. The rat CCC, mouse M3, and mouse M4 cell lines were grown in DMEM supplemented with 10% fetal bovine serum and penicillin-streptomycin. The rat CCC cells were previously established and published on in our previous study 32. The mouse CCA cells were isolated from Alb-Cre/Kras^G12D^/p53^Lox/Lox^ transgenic mice 33. The isolated CCA cells were inoculated in subcutaneous regions of C57BL/6 mice three consecutive times to generate M2, M3, and M4 cells (Figure S1D-E). Two human GR sublines (SNU-1196-GR and SSP-25-GR) were generated in the cell culture system as previously described 34. Three murine GR sublines (CCC-GR, M3-GR, and M4-GR) were generated in the cell culture system. Those cells were grown in medium containing an IC_90_ dose of GEM for three months. After three months, the live cells were identified as GR cells. All the cell lines used in this study were tested for mycoplasma contamination and authenticated by the short tandem repeat (STR) method.

### Rat and mouse experiments

Sprague-Dawley (SD) rats were purchased from BioLASCO Taiwan Co., Ltd., and BALB/cAnN. Cg-Foxn1^nu^/CrlNarl (BALB/c nude) mice were purchased from the National Laboratory Animal Center, Taiwan. The experimental procedures were reviewed and approved by the Institutional Animal Care and Use Committee of Chang Gung Memorial Hospital (approval No: IACUC 2019011601 for rat experiments and IACUC 2020112501 for mouse experiments). For the TAA-induced spontaneous rat CCA model, the 10-week-old SD rats were fed drinking water with TAA (300 mg/L). After 30 weeks, animal positron emission tomography (PET) was performed to check for the formation of CCA in the rats. Those rats received 25 mg/kg GEM or control PBS weekly for eight weeks and 50 mg/kg GEM or control PBS weekly for another eight weeks. After sixteen weeks, the remaining CCA tumors were confirmed by animal PET and lysed, after which the candidate proteins were detected by western blotting. For the xenograft mouse model, SNU-1196-GR cells (5x10^6^) were injected into the subcutaneous tissue of 6- to 7-week-old BALB/c nude mice. When tumors reached a volume of 100 to 150 mm^3^, the mice were given 100 mg/kg capivasertib o*r* the corresponding vehicles by oral gavage five times per week or 100 mg/kg GEM weekly by intraperitoneal injection for three weeks. After three weeks, the remaining tumor tissues were lysed, and candidate protein levels were detected by western blotting.

### Patient samples

Sixty-three paraffin-embedded biopsy specimens (Table S1) for Figures 6E, 6F, 6H and 6I were pathologically confirmed CCA samples retrieved from Linkou Chang Gung Memorial Hospital, and the study was approved by the Institutional Review Board Linkou Chang Gung Memorial Hospital (IRB 202002061B0). Eleven paraffin-embedded biopsy specimens and plasma samples for Figure 6G were pathologically confirmed CCA samples retrieved from Linkou Chang Gung Memorial Hospital. This study was approved by the Institutional Review Board of Linkou Chang Gung Memorial Hospital (IRB 201901646A3). Immunohistochemistry was performed as previously described 26. In brief, a 4 μm section was incubated with an anti-MUC4 antibody (1G8, Thermo Fisher Scientific Inc. Waltham, MA) at 4 °C overnight. Visualization using the REAL EnVision Detection System (K500711, DAKO, Agilent Technologies, Inc. Santa Clara, California, USA) was performed according to the manufacturer's instructions. H scores were calculated by multiplying the intensity by the percentage of the positive area. To detect MUC4 expression in plasma samples, whole blood samples in EDTA-precoated purple-top tubes were centrifuged at 1700 xg for 15 minutes at 4 °C. The supernatants were transferred to new tubes and stored at -80 °C. MUC4 expression was detected using the Human Mucin 4 ELISA Kit (MBS040933, MyBioSource, Inc.; San Diego, California).

### Recombinant plasmids and reagents

The pCDH-GFP-MUC4 expression vector was generated by inserting full-length MUC4 beta (NM_138297) into the pCDH-CMV-MCS-EF1α-copGFP vector. GEM, MK-2206 (AKT inhibitor), and afatinib were purchased from AdooQ BioScience (Irvine, CA, US). Caspase-Glo^®^ 3/7 Reagent (G8090) was purchased from Promega Corporation (Wisconsin, United States). Capivasertib (AZD5363, AKT inhibitor) was provided by AstraZeneca plc. (Cambridge, UK).

### Virus production and infection

The pCMV-ΔR8.91, pMD.G, pLKO.1-shRNA clones (Table S2; the National RNAi Core Facility, Academia Sinica, Taiwan) or pCDH-GFP-MUC4 were co-transfected into HEK293T cells using jetPEI (Polyplus Transfection, New York, NY, USA). The virus-containing supernatants were collected at 48 and 72 hours after transfection, and lentivirus particles were collected and stored at -80°C. For viral infection, the cells were mixed with virus-containing supernatants supplemented with 8 μg/ml polybrene (Sigma‒Aldrich, St. Louis, MO). After infection with the virus for 24 hours, the infected cells were selected with 1~1.5 μg/ml puromycin for another three days. For MUC4 overexpression, GFP-positive cells were sorted by BD FACSMelody™ Cell Sorter (BD Transduction Laboratories™ Franklin Lakes, NJ).

### Cell viability assays and combination index assessment

For half-maximal inhibitory concentration (IC_50_) measurement, the cells (3-8 x10^3^) were seeded in 96-well plates overnight and then cultured in ten doses of GEM. After 72 hours, cell viability was quantified by a CCK-8 assay (Dojindo Molecular Technologies, Inc., Kumamoto, Japan) according to the manufacturer's instructions. The IC_50_ values were calculated using Prism 5 (GraphPad Software, San Diego, CA). For combination index (*CI*) determination, the cells (5-8 x10^3^) were seeded in 96-well plates overnight and then cultured in ten doses of GEM, capivasertib, MK-2206, or afatinib. After 72 hours, cell viability was quantified by a CCK-8 assay. The CI values were calculated using CompuSyn software 35. According to the user's guide, CI values were defined as follows: < 0.1, very strong synergism; 0.1-0.3, strong synergism; 0.3-0.7, synergism; 0.7-0.85, moderate synergism; 0.85-0.90, slight synergism.

### Immunoblotting

Immunoblotting was performed as previously described 34. In brief, the cells were lysed in RIPA buffer plus protease inhibitors (11697498001, Roche, Mannheim, Germany) and incubated on ice for *at least* 30 minutes. The lysates were centrifuged at 12,000 xg for 10 minutes at 4 °C, and the protein concentrations were determined via a BCA protein assay (23225, Thermo Scientific Pierce™ BCA Protein Assay, Waltham, MA). The proteins were loaded on 8%~12% SDS‒PAGE gels and then transferred onto PVDF membranes (IPVH00010, Millipore, Billerica, MA). The membranes were incubated with specific primary antibodies (Table S3) at 4 °C overnight and with secondary antibodies for 1-2 hours at room temperature. The signals were detected using UVP ChemStudio PLUS Touch (Analytik Jena AG**,** Jena, Germany). The phospho-kinase array (ARY003B, R&D Systems, Minneapolis, USA) contains 43 kinase phosphorylation sites and was used to detect the phosphorylation profiles of kinases in two pairs of GR sublines and SSP-25-GR cells transfected with shRNAs against MUC4 (shMUC4) or LacZ (shLacZ). Phospho-kinase array studies were performed according to the manufacturer's instructions, and the pixel density for each spot was detected and analyzed by a UVP ChemStudio PLUS Touch system.

### RNA Extraction and Quantitative RT‒PCR (RT‒qPCR)

RT‒qPCR was performed as previously described 34. In brief, RNA was extracted using TRIzol, and 1 μg RNA was used for reverse transcription with a HiScript I ^TM^ First Strand cDNA Synthesis Kit (Bionovas, Taipei, Taiwan) according to the manufacturer's instructions*.* The qPCR mixtures (20 µl) contained 10 µl of Fast SYBR™ Green Master Mix (4385610, Thermo Fisher Scientific, Inc., Waltham, MA) and 0.5 µM forward and reverse primers (Table S4). The product was amplified using an Applied Biosystems QuantStudio 5 Real-Time PCR System (Thermo Fisher Scientific, Inc., Waltham, MA).

### Colony formation assay

SNU-1196-GR cells (500 cells/well) were seeded in six-well plates. After 17 days, the cells were cultured in GEM, MK-2206, capivasertib, or afatinib for another four days. The cells were then fixed with cold methanol for 10 minutes and stained with 0.5% crystal violet for 10 minutes. Next, the six-well plates were washed with water, and the number of colonies was assessed using ImageJ.

### cDNA microarray

Mucin protein expression in SSP-25, SSP-25-GR, SNU-1196, and SNU-1196-GR cells was analyzed using the Affymetrix Human Genome U133A Plus 2.0 Array (Thermo Fisher Scientific, Inc., Waltham, MA).

### Statistics

The results are presented as the means ± SDs or means ± SEMs. A two-tailed independent Student's *t*-test was used to compare the continuous variables between the two groups. Progression-free survival (PFS) and overall survival (OS) rates were evaluated using the Kaplan-Meier method. Several clinicopathological variables were considered for the initial univariate analysis, which was performed using the log-rank test. The Cox proportional hazards model was applied for multivariate regression analysis. The statistical software package SPSS for Windows (SPSS version 17.0, Chicago, IL, USA) was used for statistical analysis. Differences were considered statistically significant at *P* < 0.05 for all of the tests.

## Results

### MUC4 is primarily responsible for GEM sensitivity in CCA cells

**Figure 1:**
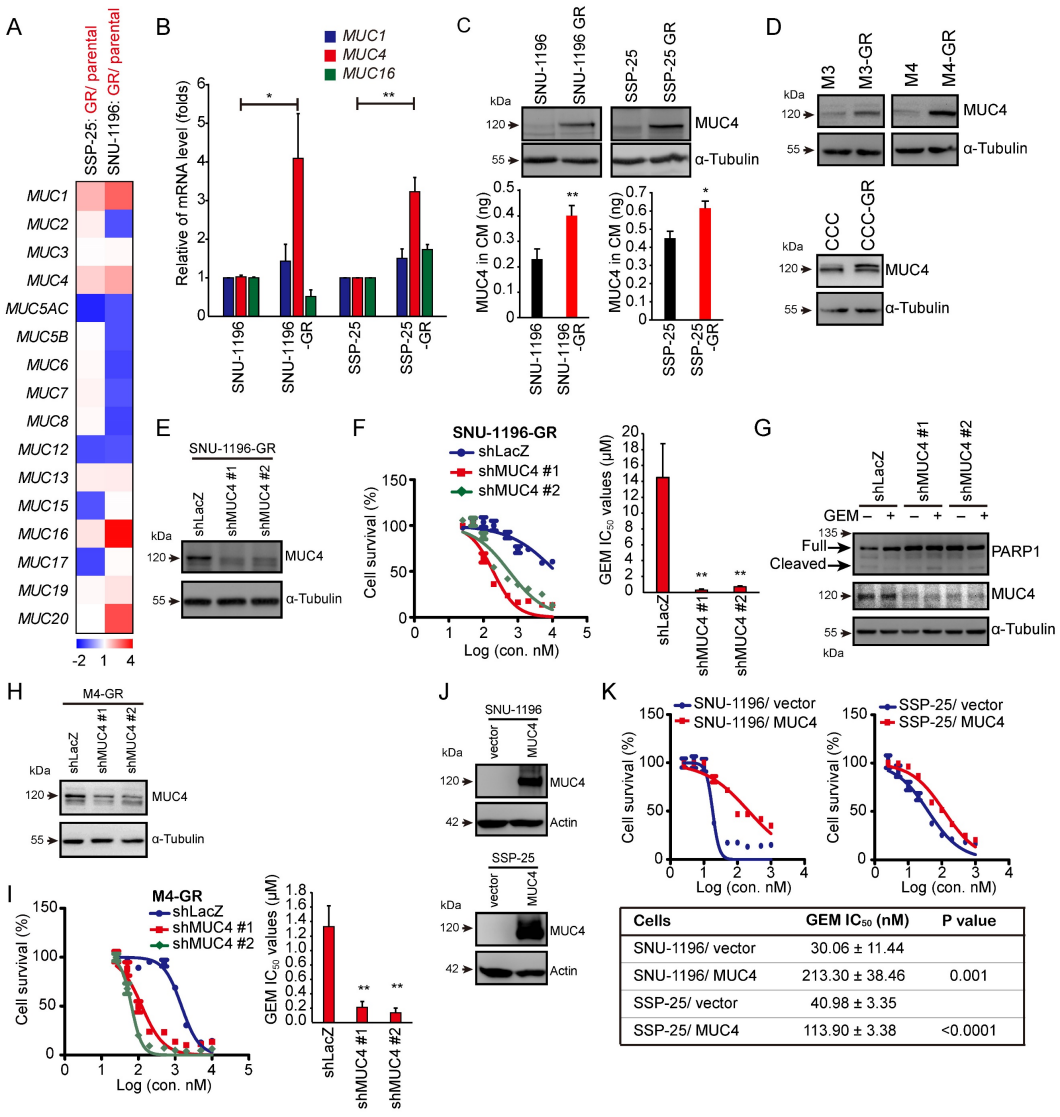
** MUC4 conferred GEM resistance in CCA cells.** (A) A heatmap showed the relative mRNA expression of *MUC* genes from resistant sublines (SNU-1196-GR and SSP-25-GR) compared with that of their *parental cells (SNU-1196 and SSP-25).* Red pixels: upregulated expression; blue pixels: downregulated expression. (B) The relative mRNA levels of* MUC1, MUC4,* and *MUC16* (n=3). The values (means ± SDs) are presented as the fold-change relative to the level in parental cells *(SNU-1196 and SSP-25)*. *, P < 0.05; **, P < 0.005 by Student's *t test*. (C) Upper: western blots showing the protein level of MUC4 in whole-cell lysates. α-Tubulin was used as the loading control. Lower: The expression of MUC4 in conditioned media (CM) was detected by an ELISA kit in human CCA cells (n=3). *, P < 0.05; **, P < 0.005 by Student's *t test*. (D) Western blots showing the protein level of MUC4 in three pairs of murine CCA sublines. α-Tubulin was used as the loading control. (E) MUC4 was depleted by shRNAs (shMUC4 #1 and #2) in SNU-1196-GR cells. Western blots showing the knockdown efficacy of SNU-1196-GR cells transfected with shRNAs specific to MUC4 (shMUC4 #1 and #2) or LacZ (shLacZ). (F) Left: cell *viability* in various concentrations of GEM. Right: The IC_50_ values (means ± SDs) were from three independent experiments. **, P < 0.005 by Student's *t-test*. (G) Western blots showing the levels of PARP1 and MUC4 in SSP-25-GR cells transfected with shRNAs specific to MUC4 (shMUC4 #1 and #2) or LacZ (shLacZ). The cells were treated with 500 nM GEM (+) or DMSO (-) for 48 hours. α-Tubulin was used as the loading control. (H) MUC4 was depleted by shRNA transfection (shMUC4 #1 and #2) in M4-GR cells. Western blots showing the knockdown efficacy in M4-GR cells transfected with shRNAs specific to MUC4 (shMUC4 #1 and #2) or LacZ (shLacZ). (I) Left: cell *viability* in various concentrations of GEM. Right: the IC_50_ values (means ± SDs) from three independent experiments. **, P < 0.005 by Student's *t-test*. (J) Western blots showing the levels of MUC4 in MUC4-overexpressing SNU-1196 and SSP-25 cells. Actin was used as the loading control. (K) Upper: cell *viability* in various concentrations of GEM. Lower: the IC_50_ values (means ± SDs) were from three independent experiments. The p values from Student's *t-test* are shown in the table.

The aim of this study was to investigate the role of mucin proteins in GEM resistance in CCA. We established two GR CCA sublines (SNU-1196-GR and SSP-25-GR) from two CCA lines (SNU-1196 and SSP-25) in vitro (Figure S1A). Compared to the parental CCA cell lines, the two GR CCA sublines exhibited reduced production of cleaved PARP1 at the same GEM dose (Figure S1B). Increased GEM IC_50_ values were also observed in the two GR CCA sublines (Figure S1C). Two pairs of sublines (SNU-1196/SNU-1196-GR and SSP-25/SSP-25-GR) were subjected to cDNA microarray analysis of mucin family gene expression. Among mucin genes, M*UC1, MUC4,* and *MUC16* showed upregulated mRNA expression in both GR sublines (Figure 1A). To confirm the results, RT‒qPCR was performed. The mRNA levels of *MUC1* and* MUC4* were increased in both GR sublines, and the increase in *MUC4* mRNA levels in resistant sublines was more evident than the increase in *MUC1* mRNA levels (Figure 1B). We further detected the protein expression of MUC4 in GR cells. MUC4 protein expression was upregulated in GR CCA sublines in whole-cell lysates and conditioned media (Figure 1C). To confirm MUC4 expression levels in GR CCA from the spontaneous CCA growth models, the mouse CCA cells and thioacetamide (TAA)-induced rat CCA cells 32, 36 were used. The mouse CCA cells (M1 in Figure S1D) were isolated from Alb-Cre/Kras^G12D^/p53^Lox/Lox^ transgenic mice 33. The isolated CCA cells were inoculated in the subcutaneous regions of C57BL/6 mice three consecutive times to generate M2, M3, and M4 cells (Figure S1D-E). Three murine GR CCA sublines (M3-GR, M4-GR, and CCC-GR) were established from three parental cell lines (M3, M4, and CCC) in vitro (Figure S1F-G). Increased MUC4 expressions were also detected in three murine GR CCA sublines (M3-GR, M4-GR, and CCC-GR; Figure 1D). We next investigated whether the expression of MUC4 in CCA cells affects GEM sensitivity. In two human GR CCA sublines, the knockdown of MUC4 decreased the GEM IC_50_ values (Figures 1E-F and S1H-I). The knockdown of MUC4 increased the activity of caspase 3 and cleaved PARP1 (Figures 1G and S1J). In the mouse GR CCA sublines, MUC4 knockdown reduced the GEM IC_50_ values (Figures 1H-I and S1K-L). Conversely, GEM IC_50_ values were upregulated in MUC4-overexpressing SNU-1196 and SSP-25 cells (Figure 1J-K), suggesting that the expression of MUC4 is inversely associated with GEM sensitivity in CCA cells.

### AKT activation is involved in MUC4-modulated GEM resistance in CCA

**Figure 2:**
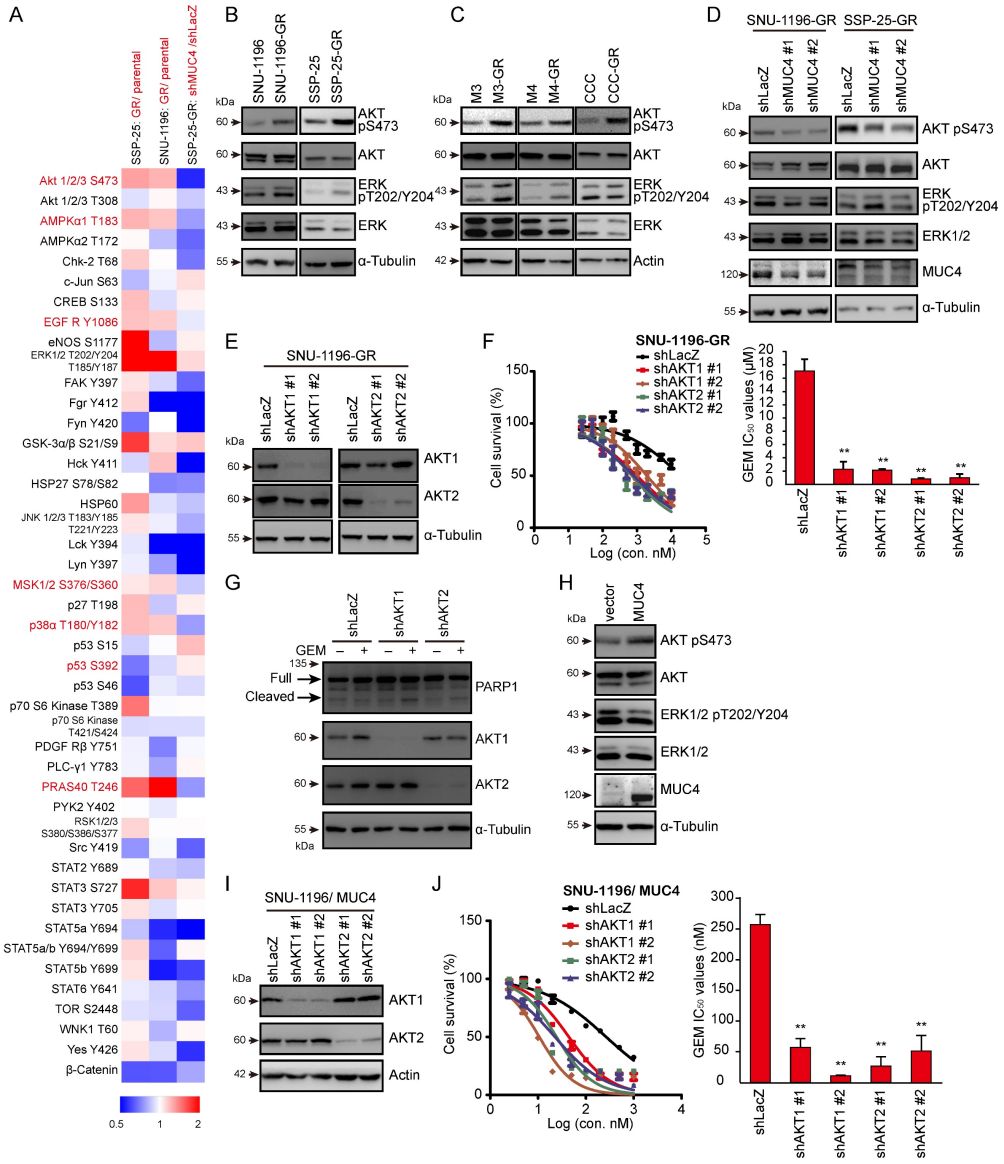
** AKT is involved in MUC4 depletion-related GEM resistance.** (A) A heatmap showed the relative changes in phosphorylation of different proteins from resistant cells (SNU-1196-GR and SSP-25-GR) versus *parental cells (SNU-1196 and SSP-25) and from* SP-25-GR cells transfected with shRNAs specific to MUC4 (shMUC4 #1) versus those transfected with shRNAs specific to LacZ (shLacZ). Red pixels: upregulated expression; blue pixels: downregulated expression. (B) Western blots showing the protein levels of phosphorylated AKT, total AKT, phosphorylated ERK, and total ERK in human CCA cell lines. α-Tubulin was used as the loading control. (C) Western blots showing the protein levels of phosphorylated AKT, total AKT, phosphorylated ERK, and total ERK in murine CCA cell lines. Actin was used as the loading control. (D) Western blots showing the protein levels of MUC4, phosphorylated AKT, total AKT, phosphorylated ERK, and total ERK in SNU-1196-GR and SSP-25-GR cells transfected with shRNAs against MUC4 (shMUC4 #1 and #2) or LacZ (shLacZ). α-Tubulin was used as the loading control. (E) Western blots showing the protein levels of AKT1 and AKT2 in SNU-1196-GR cells transfected with shRNAs against AKT1 (shAKT1 #1 and #2), AKT2 (shAKT2 #1 and #2) or LacZ (shLacZ). α-Tubulin was used as the loading control. (F) Upper: The cell *viability* in SNU-1196-GR cells transfected with shRNAs against AKT1 (shAKT1 #1 and #2), AKT2 (shAKT2 #1 and #2), or LacZ (shLacZ) in various concentrations of GEM. Lower: The IC_50_ values (means ± SDs) were from three independent experiments. **, P < 0.005 by Student's *t-test*. (G) Western blots showing the levels of PARP1 and MUC4 in SSP-25-GR cells transfected with shRNAs specific to AKT1 (shAKT1 #1 and #2), AKT2 (shAKT2 #1 and #2) or LacZ (shLacZ). The cells were treated with 500 nM GEM (+) or DMSO (-) for 48 hours. α-Tubulin was used as the loading control. (H) Western blots showing the protein levels of MUC4, phosphorylated AKT, total AKT, phosphorylated ERK, and total ERK in MUC4-overexpressing SNU-1196 cells. (I) Western blots showing the protein levels of AKT1 and AKT2 in MUC4-overexpressing SNU-1196 (SNU-1196/ MUC4) cells transfected with shRNAs against AKT1 (shAKT1 #1 and #2), AKT2 (shAKT2 #1 and #2) or LacZ (shLacZ). Actin was used as the loading control. (J) Right: The cell *viability* in MUC4-overexpressing SNU-1196 cells transfected with shRNAs against AKT1 (shAKT1 #1 and #2), AKT2 (shAKT2 #1 and #2) or LacZ (shLacZ) in various concentrations of GEM. Left: The IC_50_ values (means ± SDs) were from three independent experiments. **, P < 0.005 by Student's *t-test*.

Next, we investigated how MUC4 affects GEM sensitivity. We screened the changes in the phosphorylation of 43 human kinases via a commercial phospho-kinase array kit. Seven phospho-proteins (AKT S473, AMPKα1 T183, EGFR Y1086, MSK1/2 S376/S360, p38αT180/Y182, p53 S392, and PRAS40 T246) were upregulated in two human GR sublines and downregulated in MUC4 knockdown SSP-25-GR cells. Among those proteins, among the assessed phospho-proteins, p-AKT (S473) showed the greatest decrease upon MUC4 depletion (Figures 2A, S2A-C and Table S5). To confirm the results from the phosphokinase array, the phosphorylation of AKT was detected in five GR CCA sublines and their parental cells. The phosphorylation levels of AKT and ERK were increased in all GR sublines (Figure 2B-C). The phosphorylation of AKT but not ERK was suppressed upon the depletion of MUC4 (Figures 2D and S2D). To examine the impact of AKT on GEM sensitivity, the expression of AKT1 or AKT2 was suppressed using shRNAs. The knockdown of AKT1 or AKT2 reduced the GEM IC_50_ values in two human GR sublines (Figures 2E-F and S2E-F) and two mouse GR sublines (Figures S2H-K). AKT1 or AKT2 knockdown increased the production of cleaved PARP1 in SNU-1196-GR cells (Figure 2G) and the activity of caspase 3 in SSP-25-GR cells (Figure S2G). In MUC4-overexpressing SUN-1196 cells, AKT Ser 473 phosphorylation was enhanced (Figure 2H), indicating that AKT phosphorylation was mediated by the manipulation of MUC4 expression. Consistently, the depletion of AKT1 or AKT2 by shRNAs reduced GEM IC_50_ values in MUC4-overexpressing SUN-1196 and SSP-25 cells (Figures 2I-J and S2L-M). Taken together, these results indicate that MUC4 modulates gemcitabine sensitivity via AKT activation in CCA cells.

### Knockdown of the MUC4-associated membrane protein HER2 increased GEM sensitivity

**Figure 3:**
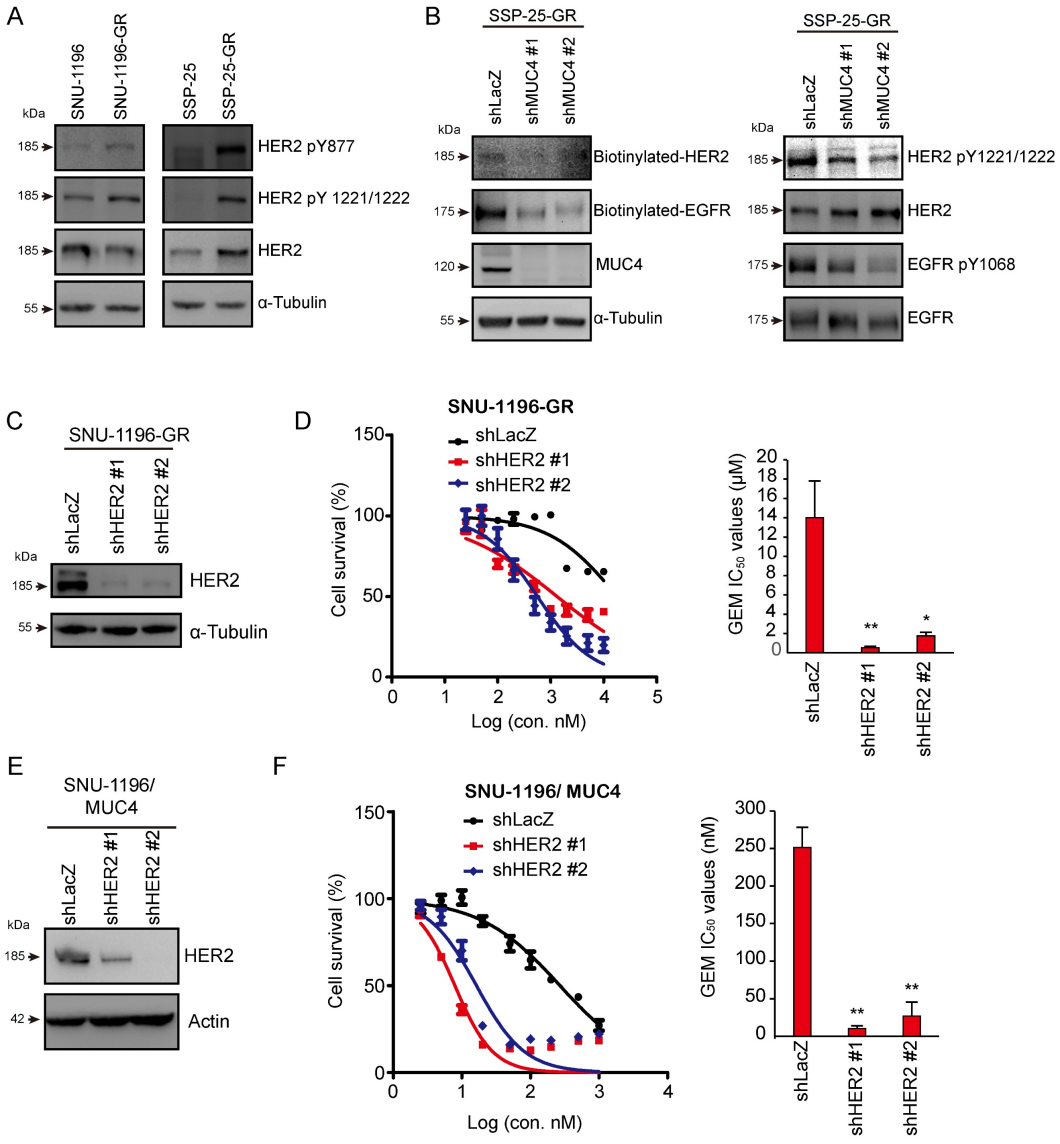
** The knockdown of HER2 reduced GEM sensitivity in GR- and MUC4-overexpressing cells.** (A) Western blots showing the protein levels of phosphorylated HER2 and total HER2 in SNU-1196, SNU-1196-GR, SSP-25, and SSP-25-GR cells. α-Tubulin was used as the loading control. (B) Western blots showing the protein levels of phosphorylated EGFR, biotinylated EGFR, total EGFR, phosphorylated HER2, biotinylated EGFR, total HER2, and MUC4 in SSP-25-GR cells transfected with shRNAs against MUC4 (shMUC4 #1 and #2) or LacZ (shLacZ). α-Tubulin was used as the loading control. (C) Western blots showing the protein level of HER2 in SNU-1196-GR cells transfected with shRNAs against HER2 (shHER2 #1 and #2) or LacZ (shLacZ). α-Tubulin was used as the loading control. (D) Right: The cell *viability* in SNU-1196-GR cells transfected with shRNAs against HER2 (shHER2 #1 and #2) or LacZ (shLacZ) in various concentrations of GEM. Left: The IC_50_ values (means ± SDs) were from three independent experiments. *, P < 0.05; **, P < 0.005 by Student's *t test*. (E) Western blots showing the protein level of HER2 in MUC4-expressing SNU-1196 cells transfected with shRNAs against HER2 (shHER2 #1 and #2) or LacZ (shLacZ). Actin was used as the loading control. (F) Left: The cell *viability* in MUC4-expressing SNU-1196 cells transfected with shRNAs against HER2 (shHER2 #1 and #2) or LacZ (shLacZ) in various concentrations of GEM. Right: The IC_50_ values (means ± SDs) were from three independent experiments. **, P < 0.005 by Student's *t test*.

Since MUC4 has been demonstrated to interact with and stabilize the HER2 protein, sustaining HER2-related signaling, including AKT activation 37, we also found that HER2 phosphorylation (HER2 pY877 and HER2 pY1221/1222) was increased in two GR sublines (Figure 3A). Phosphorylated and biotinylated EGFR and HER2 levels were reduced in MUC4-depleted SSP-25-GR cells (Figure 3B). To detect the effect of HER2 on GEM sensitivity, HER2 was depleted by shRNA transfection (Figures 3C and S3A). The knockdown of HER2 decreased the GEM IC_50_ values in the SNU-1196-GR (Figure 3D) and SSP-25-GR (Figure S3B) sublines. In MUC4-overexpressing SNU-1196 and SSP-25 cells, GEM IC_50_ values were downregulated upon HER2 knockdown (Figure 3E-F and S3C-D), suggesting that HER2 increased GEM sensitivity in GEM-resistant sublines and MUC4-overexpressing CCA cells.

### AKT inhibitors in combination with GEM or afatinib repressed cell survival in GR CCA sublines and MUC4-overexpressing cells

**Figure 4:**
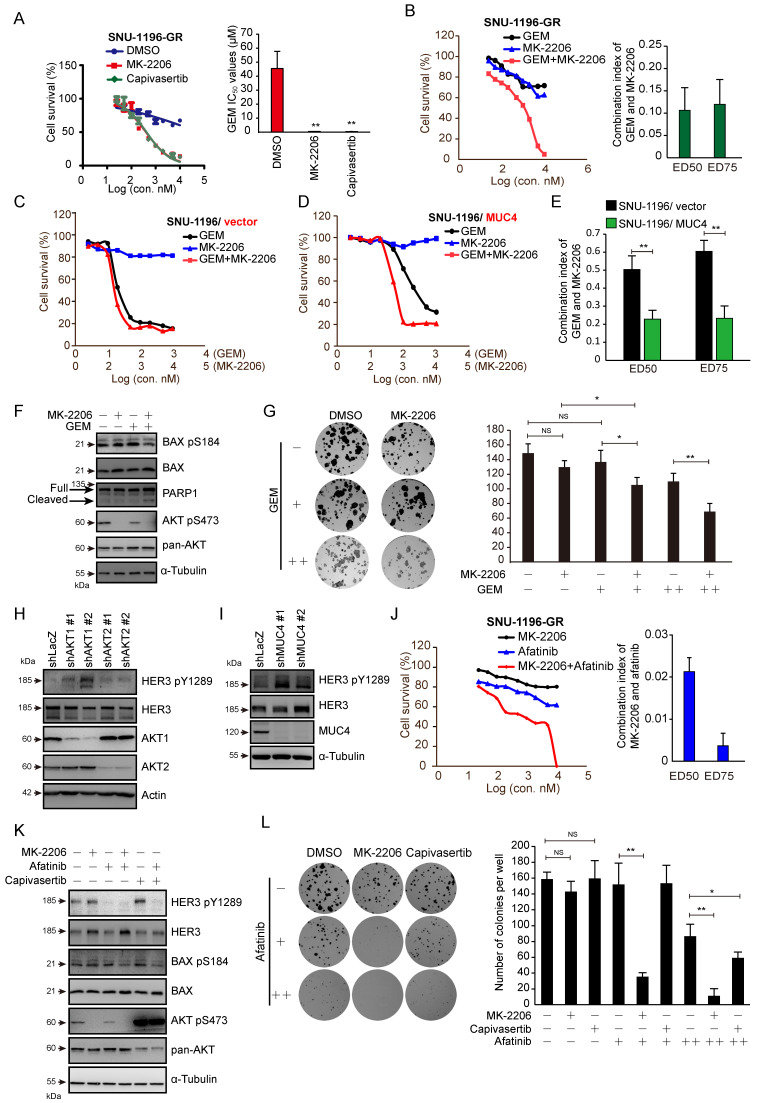
** AKT inhibitors in combination with GEM or afatinib decreased cell survival in GR sublines and MUC4-overexpressing CCA.** (A) Left: cell *viability* in various concentrations of GEM. SNU-1196-GR cells were cultured in the absence (DMSO) or presence of 2 μM MK-2206 or 2 μM capivasertib and treated with various concentrations of GEM for 72 hours. Right: The IC_50_ values (means ± SDs) were from three independent experiments. **, P < 0.005 by Student's *t-test*. (B) Left: The *viability* of SNU-1196-GR cells treated with various concentrations of MK-2206 and GEM for 72 hours. Right: The *CI* values for the combination of GEM and MK-2206 in SNU-1196-GR cells. The ED50 and ED75 values (means ± SDs) were from three independent experiments. ED, effective dose. (C), (D) The cell *viability* in SNU-1196 cells expressing the vector alone (C, SNU-1196/vector) or MUC4 (D, SNU-1196/MUC4) in various concentrations of MK-2206 and GEM for 72 hours. (E) The *CI* values for the combination of GEM and MK-2206 in SNU-1196 cells expressing the vector alone (black) or MUC4 (green). The ED50 and ED75 values (means ± SDs) were from three independent experiments. ED, effective dose. **, P < 0.005 by Student's *t*-test. (F) Western blots showing the protein levels of PARP1, phosphorylated AKT, total AKT, phosphorylated BAX, and total BAX in SNU-1196-GR cells treated with 2 μM MK-2206 (+), 1 μM GEM (+) or DMSO (-) for 48 hours. α-Tubulin was used as the loading control. (G) Left: Representative images of the colony formation assay. SUN-1196 cells (500 cells/well) were seeded in a six-well plate for 17 days and then cultured in the absence or presence of 5 μM MK-2206 (+), 50 nM GEM (+), 100 nM GEM (++), or DMSO (-) for another four days. Right: The values (means ± SDs) were from three independent experiments; * P<0.05, ** P<0.01 by Student's *t-test*. NS: *not significant*. (H) Western blots showing the protein levels of phosphorylated HER3, total HER3, AKT1 and AKT2 in SNU-1196-GR cells transfected with shRNAs against AKT1 (shAKT1 #1 and #2), AKT2 (shAKT2 #1 and #2) or LacZ (shLacZ). Actin was used as the loading control. (I) Western blots showing the protein levels of phosphorylated HER3, total HER3, and MUC4 in SNU-1196-GR cells transfected with shRNAs against MUC4 (shMUC4 #1 and #2) or LacZ (shLacZ). α-Tubulin was used as the loading control. (J) Left: The *viability* of SNU-1196-GR cells treated with various concentrations of MK-2206 and afatinib for 72 hours. Right: The *CI* values for the combination of MK-2206 and afatinib in SNU-1196-GR cells. The ED50 and ED75 values (means ± SDs) were from three independent experiments. ED, effective dose. (K) Western blots showing the protein levels of phosphorylated HER3, total HER3, phosphorylated AKT, total AKT, phosphorylated BAX, and total BAX in SNU-1196-GR cells treated with 2 μM MK-2206 (+), 2 μM capivasertib (+), 1 μM GEM (+) or DMSO (-) for 48 hours. α-Tubulin was used as the loading control. (L) Left: Representative images of the colony formation assay. SUN-1196-GR cells (500 cells/well) were seeded in a six-well plate for 17 days and then cultured in the absence or presence of 10 μM MK-2206 (+), 10 μM capivasertib (+), 2 μM afatinib (+), 5 μM afatinib (++), or DMSO (-) for another four days. Right: The values (means ± SDs) were from three independent experiments; * P<0.05, ** P<0.01 by Student's *t-test*. NS: *not significant*

We used the AKT inhibitors MK-2206 and capivasertib to confirm the role of AKT in GEM sensitivity. In the presence of the AKT inhibitor MK-2206 or capivasertib, GEM IC_50_ values were reduced in two human GR sublines (Figures 4A and S4A) and a rat GR subline (Figures S4B). The combination of MK-2206 and GEM increased cell death compared to that with MK-2206 or GEM treatment alone in SNU-1196-GR and SSP-25-GR cells (Figures 4B and S4D). We further used the *CI* method of Chou-Talalay to evaluate whether the combination of MK-2206 and GEM induces cell death in a synergistic manner 35. Strong synergistic effects (*CI*=0.1-0.3) were observed upon the combination of MK-2206 and GEM, as well as capivasertib and GEM, in SNU-1196-GR cells (Figures 4B and Figure S4C). A synergistic effect (*CI*=0.3-0.7) was observed upon combining MK-2206 and GEM in SSP-25-GR cells (Figure S4D). The cell viability upon treatment with GEM, MK-2206, or both in CCA cells expressing the vector alone or MUC4 is shown (Figures 4C-D and S4E-F). In MUC4-overexpressing SNU-1196 and SSP-25 cells, the *CI* values for the combination of MK-2206 and GEM were decreased compared to those in control cells (Figures 4E and S4G). Moreover, increased *CI* values for the combination of capivasertib and GEM were detected in MUC4-overexpressing CCA cells compared to control cells (Figure S4H-I). AKT suppresses Bax translocation to mitochondria by directly phosphorylating Bax at residue Ser 184 to inhibit apoptosis 38, 39. Bax S18 phosphorylation was suppressed, and the expression of cleaved PARP1 was increased upon the combination of MK-2206 and GEM (Figure 4F). MK-2206 repressed colony formation upon GEM treatment (Figure 4G). In several studies and trials, the combination of AKT inhibitors and other therapeutic drugs achieved greater anticancer efficiency than AKT inhibitors alone 40. Since the suppression of AKT has been demonstrated to induce a positive feedback loop related to HER3 expression and phosphorylation 41, we detected HER3 phosphorylation upon AKT or MUC4 knockdown.

The knockdown of MUC4, AKT1, or AKT2, as well as treatment with MK-2206 or capivasertib alone, enhanced the phosphorylation of HER3 (Figures 4H-I and 4K). Strong synergistic effects were observed upon the combination of MK-2206 and the pan-ErbB inhibitor afatinib in SNU-1196-GR and SSP-25-GR sublines (Figures 4J and S4J). A synergistic effect was observed upon combined application of capivasertib and afatinib in SSP-25-GR cells (Figure S4K). Bax Ser 184 phosphorylation was also repressed upon the combination of afatinib/MK-2206 and afatinib/capivasertib (Figure 4K). A decrease in colony formation caused by MK-2206 or capivasertib was detected in the presence of afatinib (Figure 4L). These results suggest that AKT inhibitors, in combination with GEM or afatinib, decrease the survival of GEM-resistant sublines and MUC4-overexpressing CCA cells.

### MUC4-AKT1 axis-mediated hENT1 impaired GEM sensitivity in CCA cells

**Figure 5:**
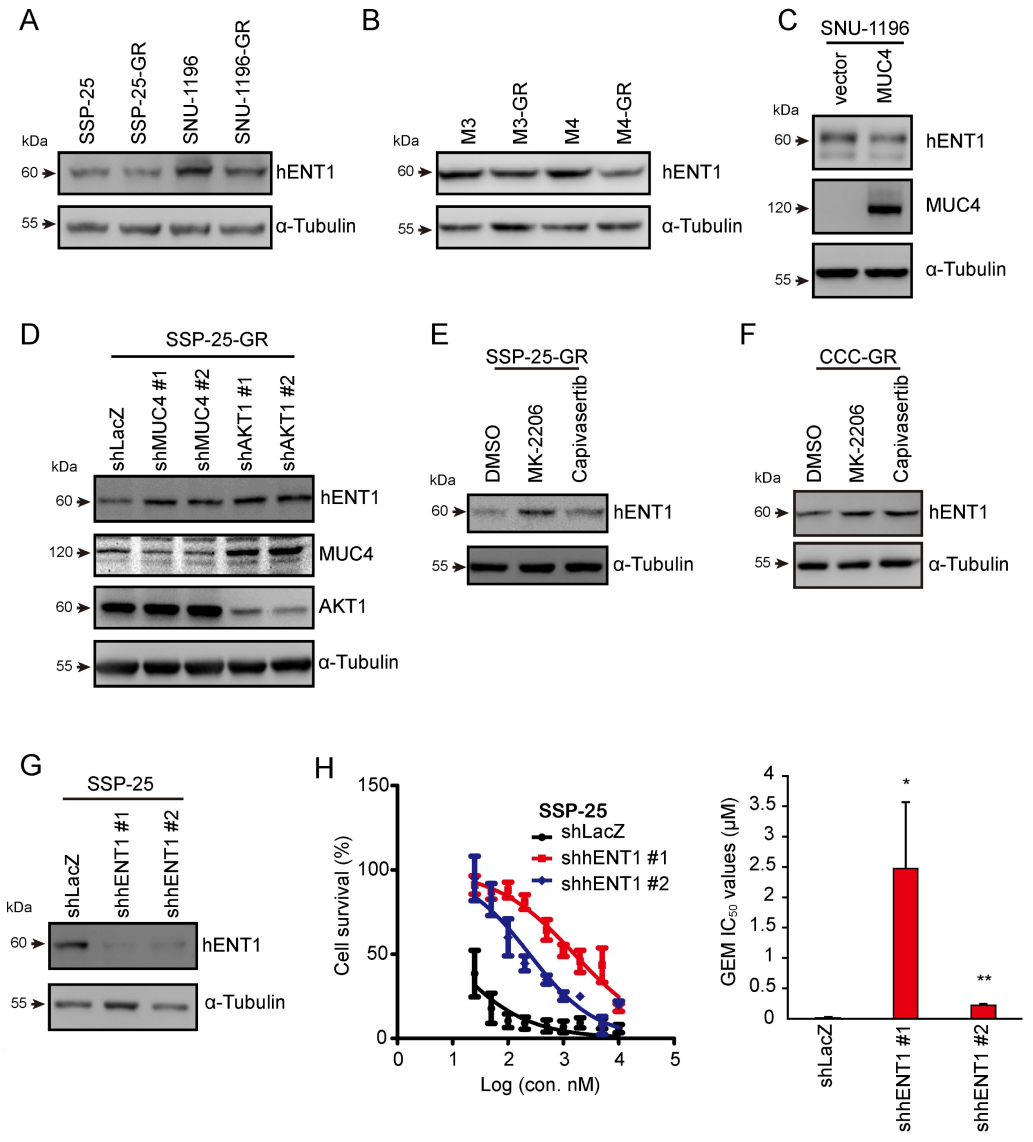
** MUC4-AKT1 axis-mediated upregulation of hENT1 decreased GEM sensitivity in CCA.** (A) Western blots showing the protein level of hENT1 in SSP-25, SSP-25-GR, SNU-1196, and SNU-1196-GR. α-Tubulin was used as the loading control. (B) Western blots showing the protein level of hENT1 in M3, M3-GR, M4, and M4-GR. α-Tubulin was used as the loading control. (C) Western blots showing the protein levels of hENT1 and MUC4 in SNU-1196 cells expressing the vector alone or in MUC4 cells. (D) Western blots showing the protein levels of hENT1, MUC4, and AKT1 in SSP-25-GR cells transfected with shRNAs against MUC4 (shMUC4 #1 and #2), AKT1 (shAKT1 #1 and #2) or LacZ (shLacZ). α-Tubulin was used as the loading control. (E) Western blots showing the protein level of hENT1 in SSP-25-GR cells receiving 2 μM MK-2206, 2 μM capivasertib, or DMSO treatment for 24 hours. α-Tubulin was used as the loading control. (F) Western blots showing the protein level of hENT1 in CCC-GR cells receiving 5 μM MK-2206, 10 μM capivasertib, or DMSO treatment for 24 hours. α-Tubulin was used as the loading control. (G) Western blots showing the protein level of hENT1 in SSP-25 cells transfected with shRNAs against hENT1 (shhENT1 #1 and #2) or LacZ (shLacZ). α-Tubulin was used as the loading control. (H) Left: The cell *viability* in SSP-25 cells transfected with shRNAs against hENT1 (shhENT1 #1 and #2) or LacZ (shLacZ) in various concentrations of GEM. Right: The IC_50_ values (means ± SDs) were from three independent experiments. *, P < 0.05; **, P < 0.005 by Student's *t-test*.

GEM is transported into cells by concentrative nucleoside transporters (hCNTs) or equilibrative nucleoside transporters (hENTs) 42, 43. GEM undergoes a series of metabolic reactions and several enzymes in the metabolic pathway. In previous studies, the dysregulation of the proteins participating in GEM metabolic pathways caused GEM resistance in pancreatic cancer 44, 45. Thus, we next investigated whether MUC4 mediates the expression of GEM metabolic genes. The mRNA levels of these genes were analyzed in two pairs of sublines and MUC4-depleted CCA cells. The mRNA expression of hENT1 (equilibrative nucleoside transporter 1), encoded by *SLC29A1,* was decreased in GR sublines and was increased in MUC4-depleted CCA cells (Figure S5A-D). The protein levels of hENT1 were reduced in four GR sublines as well as MUC4-overexpressing SNU-1196 cells (Figure 5A-C) and were increased in MUC4 knockdown SSP-25-GR cells (Figure 5D). Upon treatment with the AKT inhibitor MK-2206 or capivasertib, the expression of hENT1 was enhanced in two GR sublines (Figures 5E-F and S5E). The knockdown of AKT1, but not AKT2 or AKT3, increased hENT1 expression in GR sublines (Figures 5D and S5F-H). Moreover, the knockdown of hENT1 increased the GEM IC_50_ values in SSP-25 cells (Figure 5G-H), suggesting that hENT1 may be involved in MUC4-AKT axis-mediated GEM resistance in CCA.

### In vivo and clinical validation of the MUC4-AKT axis

**Figure 6:**
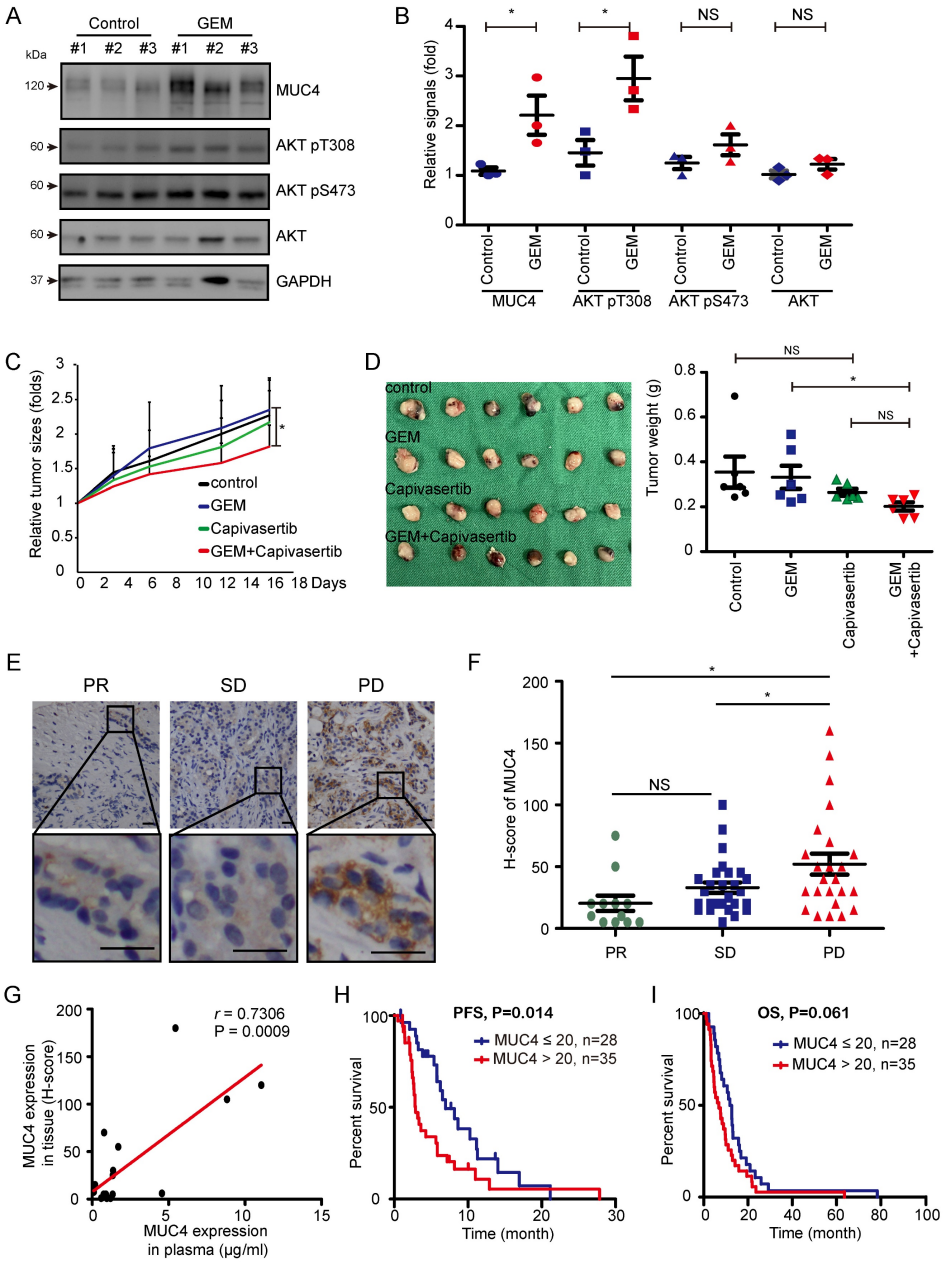
** High expression of MUC4 is associated with the poor prognosis observed in CCA patients that do not respond to GEM.** (A)Western blots showing the protein levels of MUC4, phosphorylated AKT, and total AKT in remaining rat CCA tissues after GEM or control vehicle (PBS) treatment for 16 weeks. GAPDH was used as the loading control. (B) Quantification of the relative MUC4, phosphorylated AKT, and total AKT signals in panel (A). Data represent the mean ± SEM. n=3 for each group. *P < 0.05 by Student's *t-test*. NS: *not significant.* (C), (D) The xenograft animal model treated with or without GEM and capivasertib. SNU-1196-GR cells were injected into the* subcutaneous* tissue of* BALB*/*c nude mice.* The mice were given 100 mg/kg GEM weekly by *intraperitoneal injection* and 100 mg/kg capivasertib by oral gavage five times per week for 3 weeks. After 3 weeks, the mice were sacrificed, and the relative tumor sizes (C) and tumor volumes (D) are shown. n=6 for each group. The values in panel C (means ± SEMs) are presented as fold-change relative to the tumor size at day* 0.* *P < 0.05 by Student's *t-test*. NS: *not significant.* (E) Representative images of immunohistochemical staining for MUC4*.* Scale bar = 25 μm. (F) *Distribution* of the H score for MUC4 expression from 63 GEM-based chemotherapy-treated CCA patients with partial response (PR, n=12), stable disease (SD, n=27), or progressive disease (PD, n=24). Data represent the mean ± SEM. *P < 0.05 by Student's *t-test*. NS: *not significant*. (G) Association between MUC4 expression in paraffin-embedded specimens and plasma samples from 17 patients. The *Pearson* correlation coefficient *r* and P values are shown in the panel. (H), (I) Kaplan‒Meier plots of the progression-free survival (PFS; H) and overall survival (OS; I) of CCA cancer patients with high MUC4 expression (n=35) and low expression (n=28)*. The* P values shown in each panel were determined by the *log*-*rank test.*

Finally, we examined the impact of the MUC4-AKT axis on GEM resistance in vivo. A TAA-induced spontaneous rat CCA model was used 36. SD rats were administered TAA for 30 weeks, and GEM was then injected into the rats every week for sixteen consecutive weeks (Figure S6A). After sixteen weeks, MUC4 expression and AKT phosphorylation were detected in TAA-induced rat CCA tissues. MUC4 expression and AKT Thr308 phosphorylation were upregulated in the remaining tumor tissues (Figure 6A-B and Table S6). The GR subline SNU-1196-GR was injected into the subcutaneous tissue of BALB/c nude mice. The combination of capivasertib and GEM compared to GEM alone significantly impaired tumor growth in vivo (Figure 6C-D). In SNU-1196-GR-derived tumor tissues, the combination of capivasertib and GEM enhanced hENT1 expression and reduced BAX Ser184 phosphorylation (Figure S6B-D and Table S7). In CCA patients, the expression of MUC4 in GEM-based chemotherapy-treated patients with partial response (PR) or stable disease (SD) was lower than its expression in GEM-based chemotherapy-treated patients with progressive disease (PD; Figure 6E-F). The increased MUC4 expression in the patients' plasma positively correlated with the increased expression in the paraffin-embedded specimens (Figure 6G). High expression of MUC4 in CCA patients was associated with poor progression-free survival (PFS) but not overall survival (OS; Figure 6I-H). In univariate analysis, CCA patients undergoing chemotherapy with high expressions of MUC4 or lung metastasis had an inferior PFS compared with that of CCA patients with low MUC4 expression or without lung metastasis (Table 1). In multivariate analysis, CCA patients with bone metastasis or peritoneum metastasis had an inferior OS (Table S8).

## Discussion

In this study, we demonstrated the role of the MUC4-HER2-AKT axis in GR CCA in vitro and in vivo. First, MUC4 was identified using microarray, upregulated cell lysates, and conditioned medium in GR sublines. Next, AKT activation mediated by MUC4 was demonstrated by a phospho-kinase array. The combination of AKT inhibitors with GEM or afatinib overcame GEM resistance in CCA cells. MUC4 sustained EGFR and HER2 phosphorylation to modulate AKT activation, resulting in suppression of BAX-mediated apoptosis and hENT1 expression. Finally, we clarified the role of the MUC4-AKT axis in GEM resistance in a xenograft model and validated the impact of MUC4 immunostaining on the GEM response in CCA patients.

In normal cholangiocytes, high expression of MUC3, MUC6, and MUC5B was found, but low expression of MUC1, MUC5AC, and MUC2 and no expression of MUC4 and MUC7 were detected 46. In CCAs, the expression levels of MUC1, MUC5AC, and MUC6 were significantly increased 47. High MUC1 expression was significantly associated with poor survival, high MUC5AC expression was frequently detected in advanced patients, and high MUC6 expression was significantly related to well-differentiated CCAs 48. In BTC carcinogenesis, MUC4 expression in patient tissue specimens and bile samples was significantly increased 49. In gallbladder carcinoma, high MUC4 expression was significantly associated with poor survival 50. However, the role of MUC4 in drug resistance in BTC has not yet been uncovered. In this study, we discovered a new role for MUC4 in GEM resistance in CCA. In our results, the increased MUC4 expression enhanced AKT-mediated anti-apoptosis signals, resulting in GEM resistance in CCA. Moreover, MUC4 is a secreted mucin protein, and MUC4 expression can be detected in patient plasma. This study may provide rapid and valuable information for the clinical diagnosis of GEM resistance in advanced BTC patients.

Mucin proteins have been demonstrated to cause drug resistance in other cancers by acting as barriers to drugs or antibodies, facilitating resistance to apoptosis, and altering drug metabolism, cell stemness, and epithelial-mesenchymal transition (EMT) 51. MUC1 and MUC4 are often involved in drug resistance. In pancreatic cancer, MUC4 mediates GEM resistance by suppressing hCNT1 expression and activating ErbB2 and ERK 30, 52. Here, we demonstrated that MUC4 induces GEM resistance in CCA by modulating ErbB proteins, including EGFR, HER2, and HER3. Moreover, MUC4-promoted AKT phosphorylation induces antiapoptotic BAX Ser 184 phosphorylation and inhibits hENT1 expression in GR CCA cells. Finally, we provided a potential combination therapy of an AKT inhibitor and GEM or afatinib to overcome GEM resistance.

There are many preclinical studies on AKT inhibitor combination strategies in different cancers 41, 53-56. Since AKT inhibitor monotherapies usually induce other feedback loops to cause resistance, AKT inhibitor combination therapies are more effective than AKT inhibitors alone 41, 57, 58. This study confirmed that the AKT inhibitor alone does not suppress cell survival, BAX Ser 184 phosphorylation, or colony formation in GR CCAs (Figure 4). In CCA cells, treatment with an AKT inhibitor or knockdown of AKT1 or AKT2 induced HER3 expression or activation (Figure 4H and 4K), and the AKT inhibitor combined with afatinib significantly induced cell death and repressed BAX Ser 184 phosphorylation and colony formation (Figure 4J and 4L). In addition, the combination of GEM and AKT inhibitors also effectively impaired cell survival (Figure 4B-G). In vivo, capivasertib-GEM combination therapy suppressed the development of tumors from CCA-GR cells (Figure 6B).

In conclusion, this study demonstrated that MUC4 mediates GEM resistance via HER2/AKT signaling in vitro and in vivo. In line with these results, combined treatment with an AKT inhibitor and GEM could overcome GEM resistance in CCA. Notably, the expression of MUC4 can be detected in the plasma and is correlated well with the relative expression of MUC4 in paraffin-embedded specimens in CCA patients, providing the feasibility for prompt clinical monitoring of GEM response. Further prospective trials are needed to confirm the potential utility of our findings.

## Supplementary Material

Supplementary figures and tables.

## Figures and Tables

**Table 1:** Univariate and multivariate analysis of prognostic factors (PFS)

Factors	Median (months)	95% C.I. of median	P value	Hazard ratios	95% C.I. of HR	P value
Age (years)			0.997	-		
≦65 (n=38)	5.85	3.21-8.49				
>65 (n=25)	5.65	1.54-9.76				
Gender			0.938	-		
Male (n=32)	4.27	0.63-7.91				
Female (n=31)	5.78	3.76-7.80				
Performance score			0.535	-		
0/1 (n=54)	5.65	3.29-8.02				
2 (n=9)	6.24	1.84-10.65				
MUC4 expression			**0.014**			
≦20 (n=28)	6.97	4.34-9.59		Reference		
>20 (n=35)	2.89	2.26-3.52		2.27	1.15-4.49	**0.018**
Lung meta			**0.002**			
Yes (n=12)	3.02	2.48-3.56		2.53	1.15-5.55	**0.021**
No (n=51)	6.24	4.71-7.77		Reference		
Liver meta			0.24	-		
Yes (n=22)	3.15	2.06-4.25				
No (n=41)	5.85	4.88-6.82				
Bone meta			0.168	-		
Yes (n=6)	3.02	2.11-3.94				
No (n=57)	5.78	3.87-7.70				
Peritoneum meta			0.225	-		
Yes (n=9)	3.35	0.01-7.09				
No (n=54)	5.65	3.67-7.63				
Distant LNs meta			0.718	-		
Yes (n=9)	3.61	0.01-8.03				
No (n=54)	5.65	3.97-7.33				
Best response			**<0.0001**			
PR (n=12)	11.01	9.77-12.24		Reference		
SD (n=27)	6.97	5.18-8.75		0.95	0.38-2.38	0.915
PD (n=24)	2.4	1.97-2.83		21.7	7.43-63.33	**<0.0001**
