# Supplementary material for: Mucin 4 Confers Gemcitabine Resistance and an Unfavorable Prognosis in Patients with Cholangiocarcinoma via AKT Activation
Source: Int J Biol Sci. 2023 May 21;19(9):2772–86. doi: 10.7150/ijbs.79126 (PMC10266071; doi:10.7150/ijbs.79126)
Supplement: Supplementary file 1 — Supplementary figures and tables. [file ijbsv19p2772s1.pdf]

Supplementary Figures

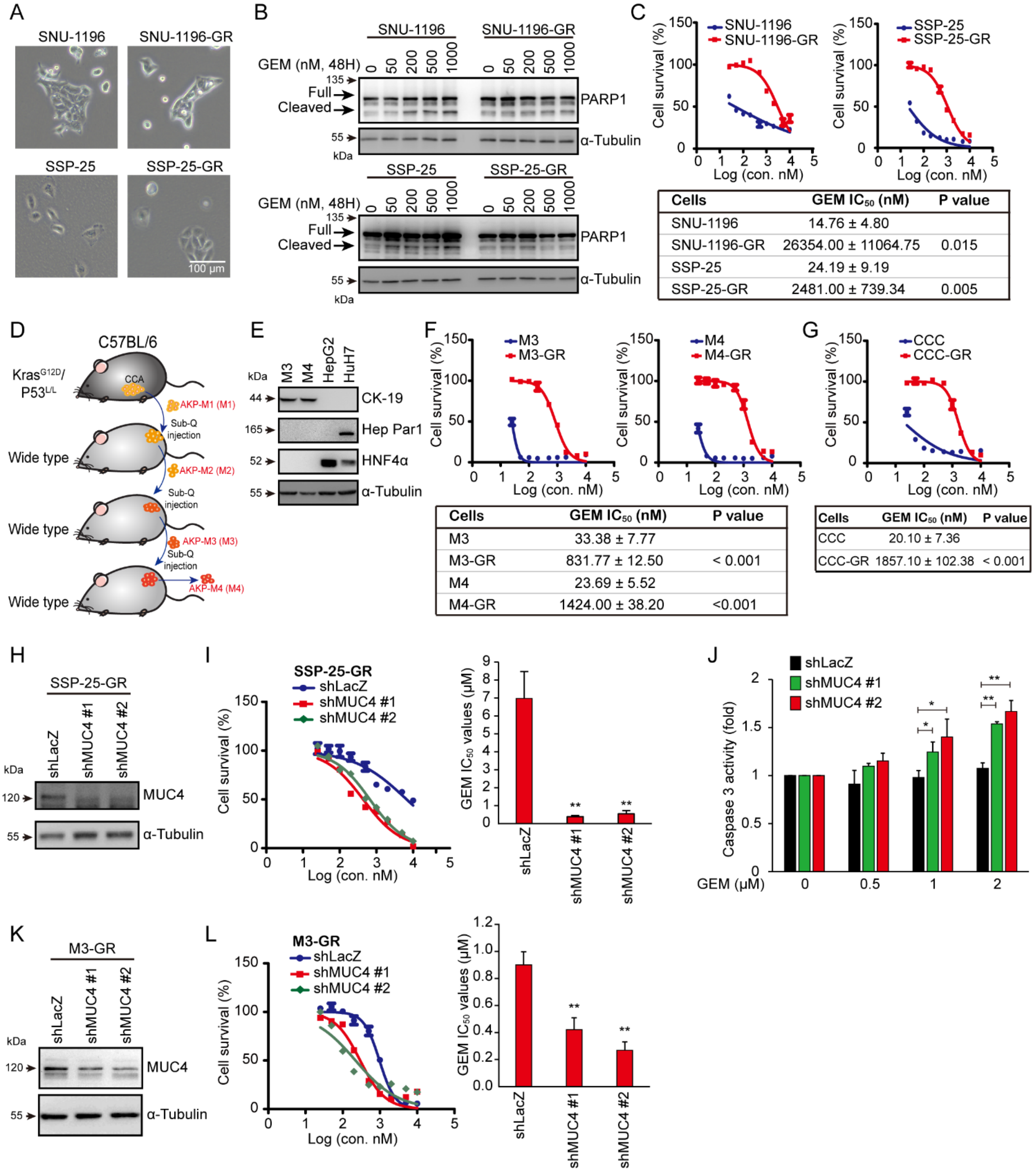

Supplementary Figure 1. Description of GR sublines and the effects of MUC4 knockdown on GEM sensitivity

(A) Representative phase-contrast images of the GR sublines (SNU-1196-GR and SSP-25-GR) and their parental cells (SNU-1196 and SSP-25).

(B) Western blots showing the levels of PARP1 in human CCA cells treated with various concentrations of GEM. α-Tubulin was used as the loading control.

(C) Upper: cell viability in the presence of various concentrations of GEM. Lower: the IC_{50} values (means ± SDs) of human CCA cells were from three independent experiments. The p values from

Student's *t tests* are shown in the table.

(D) A schematic showing the establishment of mouse M1 (AKP-M1), M2 (AKP-M2), M3 (AKP-M3), and M4 (AKP-M4) CCA cells.

(E) Western blots showing the levels of CK-19, Hep Par-1, and HNF4. α-Tubulin was used as the loading control.

(F) Upper: cell viability in the presence of various concentrations of GEM. Lower: the IC_{50} values (means ± SDs) in mouse CCA cells were from three independent experiments. The p values from Student's *t tests* are shown in the table.

(G) Upper: cell viability in the presence of various concentrations of GEM. Lower: the IC_{50} values (means ± SDs) in rat CCA cells were from three independent experiments. The p values from Student's *t tests* are shown in the table.

(H) MUC4 was depleted by shRNA transfection (shMUC4 #1 and #2) in SSP-25-GR cells. Western blots showing the knockdown efficacy in SSP-25-GR cells transfected with shRNAs specific to MUC4 (shMUC4 #1 and #2) or LacZ (shLacZ).

(I) Left: cell viability in the presence of various concentrations of GEM. Right: The IC_{50} values (means ± SDs) were from three independent experiments. **, $P < 0.005$ by Student's *t test*.

(J) Caspase-3 activity was examined using the Caspase-Glo® 3/7 assay system. Quantification of caspase-3 activity in SSP-25-GR cells transfected with shRNAs specific to MUC4 (shMUC4 #1 and #2) or LacZ (shLacZ). The cells were treated with GEM for 24 hours. The values (means ± SDs) from three independent experiments are expressed as a percentage relative to the values in cells without GEM treatment. * $P<0.05$, ** $P<0.01$ by Student's *t test*.

(K) MUC4 was depleted with shRNAs (shMUC4 #1 and #2) in M3-GR cells. Western blots showing the knockdown efficacy in M3-GR cells transfected with shRNAs specific to MUC4 (shMUC4 #1 and #2) or LacZ (shLacZ).

(L) Left: cell viability in the presence of various concentrations of GEM. Right: the IC_{50} values (means ± SDs) were from three independent experiments. **, $P < 0.005$ by Student's *t test*.

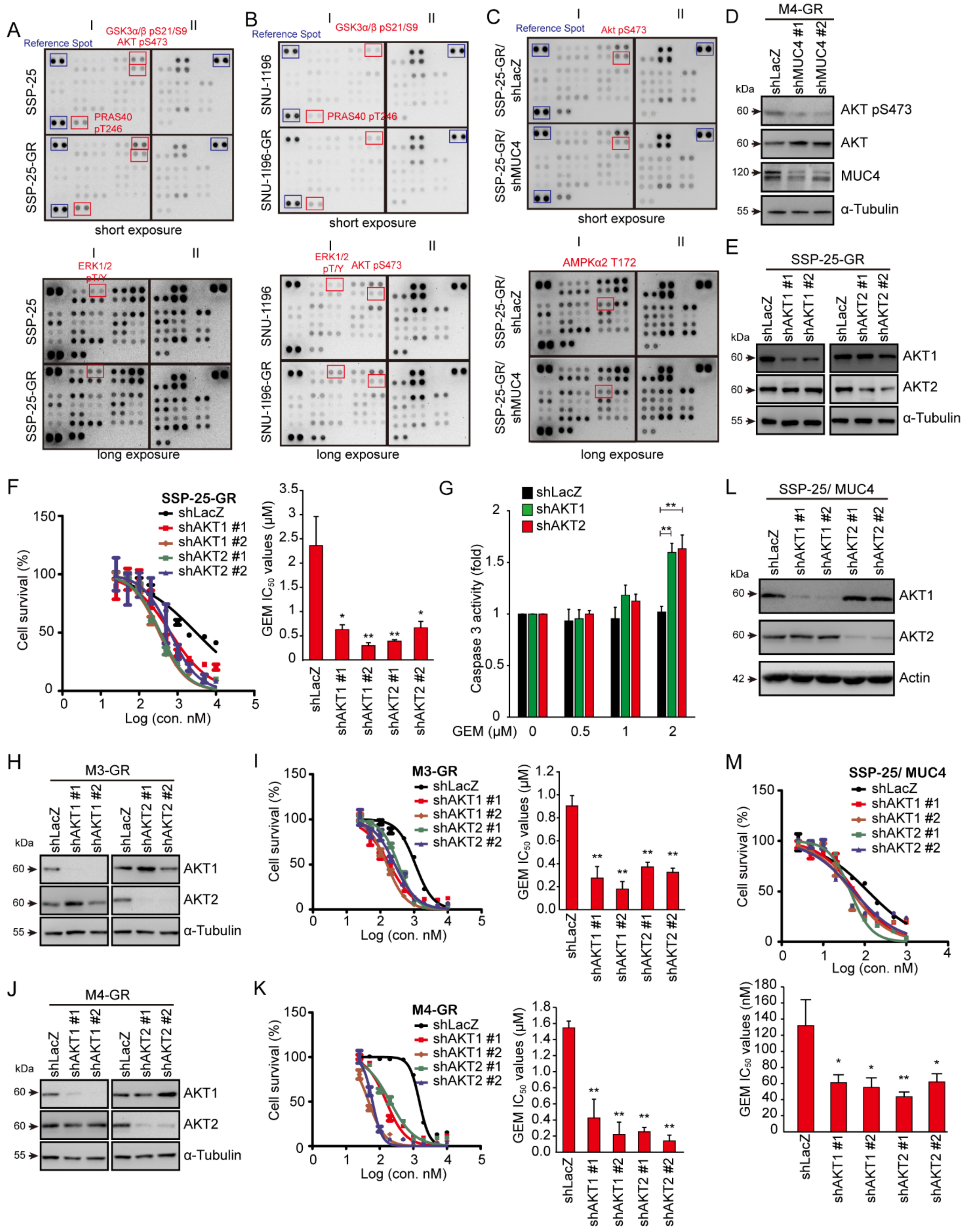

Supplementary Figure 2. Knockdown of AKT impaired GEM resistance

(A)–(C) Whole-cell lysates were prepared from SNU-1196 (A), SNU-1196-GR (A), SSP-25 (B), SSP-25-GR (B), and SSP-25-GR cells transfected with shMUC4 or shLacZ (C) and hybridized with a phosphokinase array kit.

(D) Western blots showing the protein levels of MUC4, phosphorylated AKT, and total AKT in

M4-GR cells transfected with shRNAs against MUC4 (shMUC4 #1 and #2) or LacZ (shLacZ). α-Tubulin was used as the loading control.

(E) Western blots showing the protein levels of AKT1 and AKT2 in SSP-25-GR cells transfected with shRNAs against AKT1 (shAKT1 #1 and #2), AKT2 (shAKT2 #1 and #2) or LacZ (shLacZ). α-Tubulin was used as the loading control.

(F) Left: the cell viability in SSP-25-GR cells transfected with shRNAs against AKT1 (shAKT1 #1 and #2), AKT2 (shAKT2 #1 and #2), or LacZ (shLacZ) in the presence of various concentrations of GEM. Right: The IC_{50} values (means ± SDs) were from three independent experiments. *, $P < 0.05$; **, $P < 0.005$ by Student's *t test.*

(G) Caspase-3 activity was examined using the Caspase-Glo® 3/7 assay system. Quantification of caspase-3 activity in SSP-25-GR cells transfected with shRNAs specific to AKT (shAKT1 and shAKT2) or LacZ (shLacZ). The cells were treated with GEM for 24 hours. The values (means ± SDs) from three independent experiments are expressed as a percentage relative to those in cells without GEM treatment (0 μM). ** $P<0.01$ by Student's *t test.*

(H) Western blots showing the protein levels of AKT1 and AKT2 in M3-GR cells transfected with shRNAs against AKT1 (shAKT1 #1 and #2), AKT2 (shAKT2 #1 and #2) or LacZ (shLacZ). α-Tubulin was used as the loading control.

(I) Left: cell viability in M3-GR cells transfected with shRNAs against AKT1 (shAKT1 #1 and #2), AKT2 (shAKT2 #1 and #2), or LacZ (shLacZ) in the presence of various concentrations of GEM for 72 hours. Right: the IC_{50} values (means ± SDs) were from three independent experiments. *, $P < 0.05$; **, $P < 0.005$ by Student's *t test.*

(J) Western blots showing the protein levels of AKT1 and AKT2 in M4-GR cells transfected with shRNAs against AKT1 (shAKT1 #1 and #2), AKT2 (shAKT2 #1 and #2) or LacZ (shLacZ). α-Tubulin was used as the loading control.

(K) Left: cell viability in M4-GR cells transfected with shRNAs against AKT1 (shAKT1 #1 and #2), AKT2 (shAKT2 #1 and #2), or LacZ (shLacZ) in the presence of various concentrations of GEM for 72 hours. Right: the IC_{50} values (means ± SDs) were from three independent experiments. *, $P < 0.05$; **, $P < 0.005$ by Student's *t test.*

(L) Western blots showing the protein levels of AKT1 and AKT2 in MUC4-overexpressing SSP-25 cells transfected with shRNAs against AKT1 (shAKT1 #1 and #2), AKT2 (shAKT2 #1 and #2) or LacZ (shLacZ). Actin was used as the loading control.

(M) Left: cell viability in MUC4-overexpressing SSP-25 cells transfected with shRNAs against AKT1 (shAKT1 #1 and #2), AKT2 (shAKT2 #1 and #2) or LacZ (shLacZ) in the presence of various concentrations of GEM for 72 hours. Right: the IC_{50} values (means ± SDs) were from three independent experiments. *, $P < 0.05$; **, $P < 0.005$ by Student's *t test.*

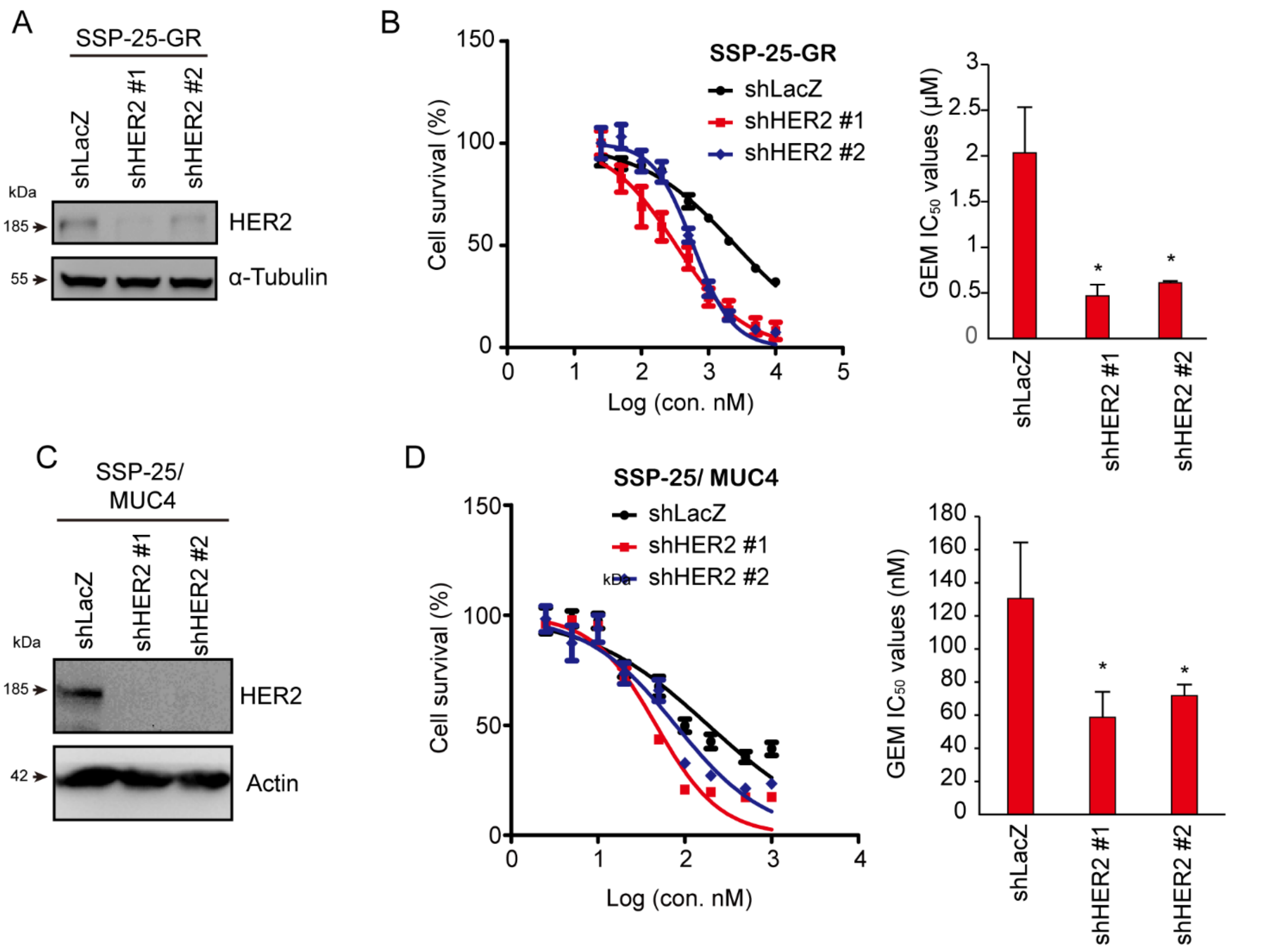

Supplementary Figure 3. HER2 knockdown increased GEM IC_{50} values

(A) Western blots showing the protein level of HER2 in SSP-25-GR cells transfected with shRNAs against HER2 (shHER2 #1 and #2) or LacZ (shLacZ). α-Tubulin was used as the loading control.

(B) Left: the cell viability in SSP-25-GR cells transfected with shRNAs against HER2 (shHER2 #1 and #2) or LacZ (shLacZ) in the presence of various concentrations of GEM. Right: the IC_{50} values (means ± SDs) were from three independent experiments. *, $P < 0.05$ by Student's *t test*.

(C) Western blots showing the protein level of HER2 in MUC4-expressing SSP-25 cells transfected with shRNAs against HER2 (shHER2 #1 and #2) or LacZ (shLacZ). α-Tubulin was used as the loading control.

(D) Left: the cell viability in MUC4-expressing SSP-25 cells transfected with shRNAs against HER2 (shHER2 #1 and #2) or LacZ (shLacZ) in the presence of various concentrations of GEM. Right: The IC_{50} values (means ± SDs) were from three independent experiments. *, $P < 0.05$ by Student's *t test*.

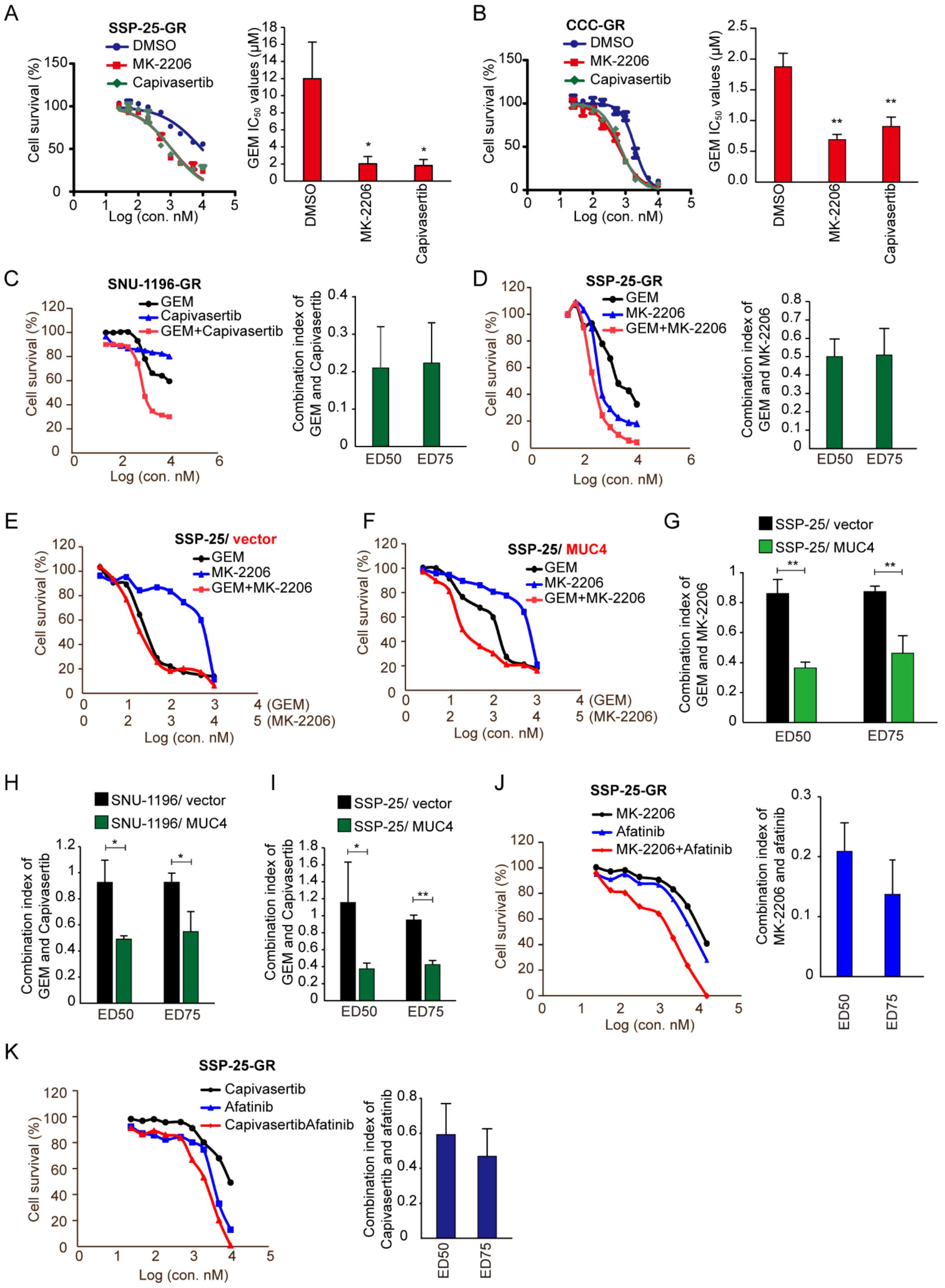

Supplementary Figure 4. The combination of AKT inhibitors and GEM or afatinib repressed cell survival

(A) Left: cell viability in the presence of various concentrations of GEM. SSP-25-GR cells were cultured in the absence (DMSO) or presence of 2 μM MK-2206 or 2 μM capivasertib and treated with various concentrations of GEM for 72 hours. Right: the IC_{50} values (means ± SDs) were from three independent experiments. *, $P < 0.05$ by Student's *t test*.

(B) Left: cell viability in the presence of various concentrations of GEM. CCC-GR cells were cultured in the absence (DMSO) or presence of 5 μM MK-2206 or 10 μM capivasertib and treated with various concentrations of GEM for 72 hours. Right: the IC_{50} values (means ± SDs) were from three independent experiments. **, $P < 0.005$ by Student's *t test*.

(C) Left: the viability of SNU-1196-GR cells treated with various concentrations of capivasertib and GEM for 72 hours. Right: the *CI* values for the combination of GEM and capivasertib in SNU-1196-GR cells. The ED50 and ED75 values (means ± SDs) were from three independent experiments. ED, effective dose.

(D) Left: the viability of SSP-25-GR cells treated with various concentrations of MK-2206 and GEM for 72 hours. Right: the *CI* values for the combination of GEM and MK-2206 in SSP-25-GR cells. The ED50 and ED75 values (means ± SDs) were from three independent experiments. ED, effective dose.

(E) , (F) The viability of SSP-25 cells expressing the vector alone (E, SSP-25/vector) or MUC4 (F, SSP-25/MUC4) in the presence of various concentrations of MK-2206 and GEM for 72 hours.

(G) The *CI* values for the combination of GEM and MK-2206 in SSP-25 cells expressing the vector alone (black) or MUC4 (green). The ED50 and ED75 values (means ± SDs) were from three independent experiments. ED, effective dose. **, $P < 0.005$ by Student's *t test*.

(H), (I) The *CI* values for the combination of GEM and capivasertib in SNU-1196 (H) or SSP-25 (I) cells expressing the vector alone (black) or MUC4 (dark green). The ED50 and ED75 values (means ± SDs) were from three independent experiments. ED, effective dose. *, $P < 0.05$; **, $P < 0.005$ by Student's *t test*.

(J) Left: viability of SSP-25-GR cells treated with various concentrations of MK-2206 and afatinib for 72 hours. Right: the *CI* values for the combination of MK-2206 and afatinib in SSP-25-GR cells. The ED50 and ED75 values (means ± SDs) were from three independent experiments. ED, effective dose.

(K) Left: viability of SSP-25-GR cells treated with various concentrations of MK-2206 and capivasertib for 72 hours. Right: the *CI* values for the combination of MK-2206 and capivasertib in SSP-25-GR cells. The ED50 and ED75 values (means ± SDs) were from three independent experiments. ED, effective dose.

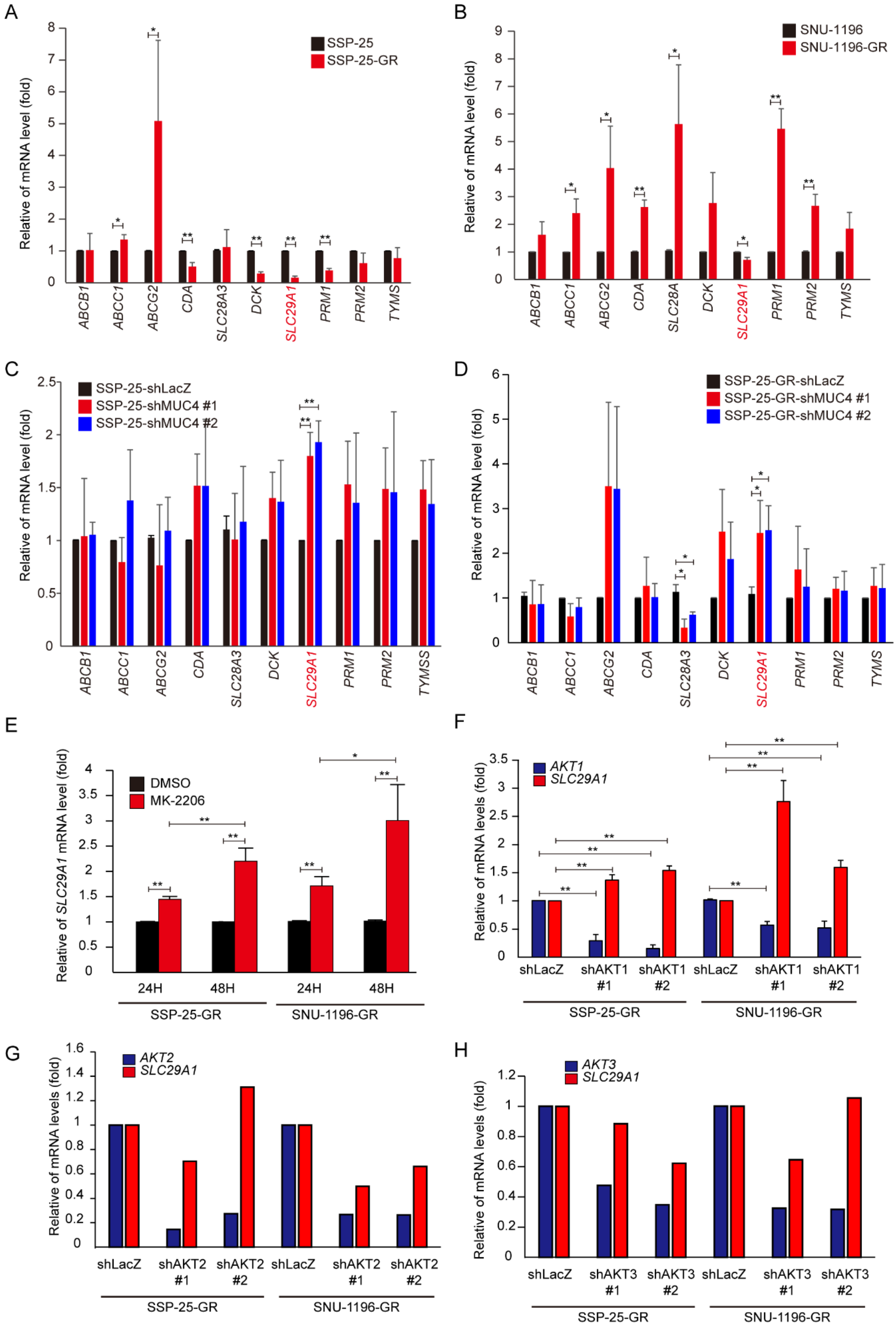

Supplementary Figure 5. *SLC29A1* expression was modulated in human GR CCA cells and MUC4/AKT1-depleted CCA cells

(A), (B) RT–qPCR to screen the expression of GEM metabolism genes in SSP-25 and SSP-25-GR

cells (A) and SNU-1196 and SNU-1196-GR cells (B). The values (means ± SDs) from three independent experiments are presented as the fold change relative to the level of SSP-25 (A) or SNU-1196 (B). *, $P < 0.05$; **, $P < 0.005$ by Student's *t test.*

(C), (D) RT–qPCR to screen the expression of GEM metabolism genes in SSP-25 (C) or SSP-25-GR (D) cells transfected with shRNAs against MUC4 (shMUC4 #1 and #2) or LacZ (shLacZ). The values (means ± SDs) from three independent experiments are presented as the fold change relative to the level of LacZ knockdown cells. *, $P < 0.05$; **, $P < 0.005$ by Student's *t test.*

(E) The relative mRNA level of *SLC29A1* in 2 μM MK-2206-treated SSP-25-GR and SNU-1196-GR cells. The values (means ± SDs) from three independent experiments are presented as the fold-change relative to the level in cells receiving DMS treatment. *, $P < 0.05$; **, $P < 0.005$ by Student's *t test.*

(F)–(H) The relative mRNA levels of *SLC29A1, AKT1* (F, n=3), *AKT2* (G, n=1), and *AKT3* (H, n=1) in SSP-25-GR and SNU-1196-GR cells transfected with shRNAs against AKT (shAKT #1 and #2). The values (means ± SDs) are presented as the fold-change relative to the levels in the cells transfected with shRNAs against LacZ (shLacZ). **, $P < 0.005$ by Student's *t test.*

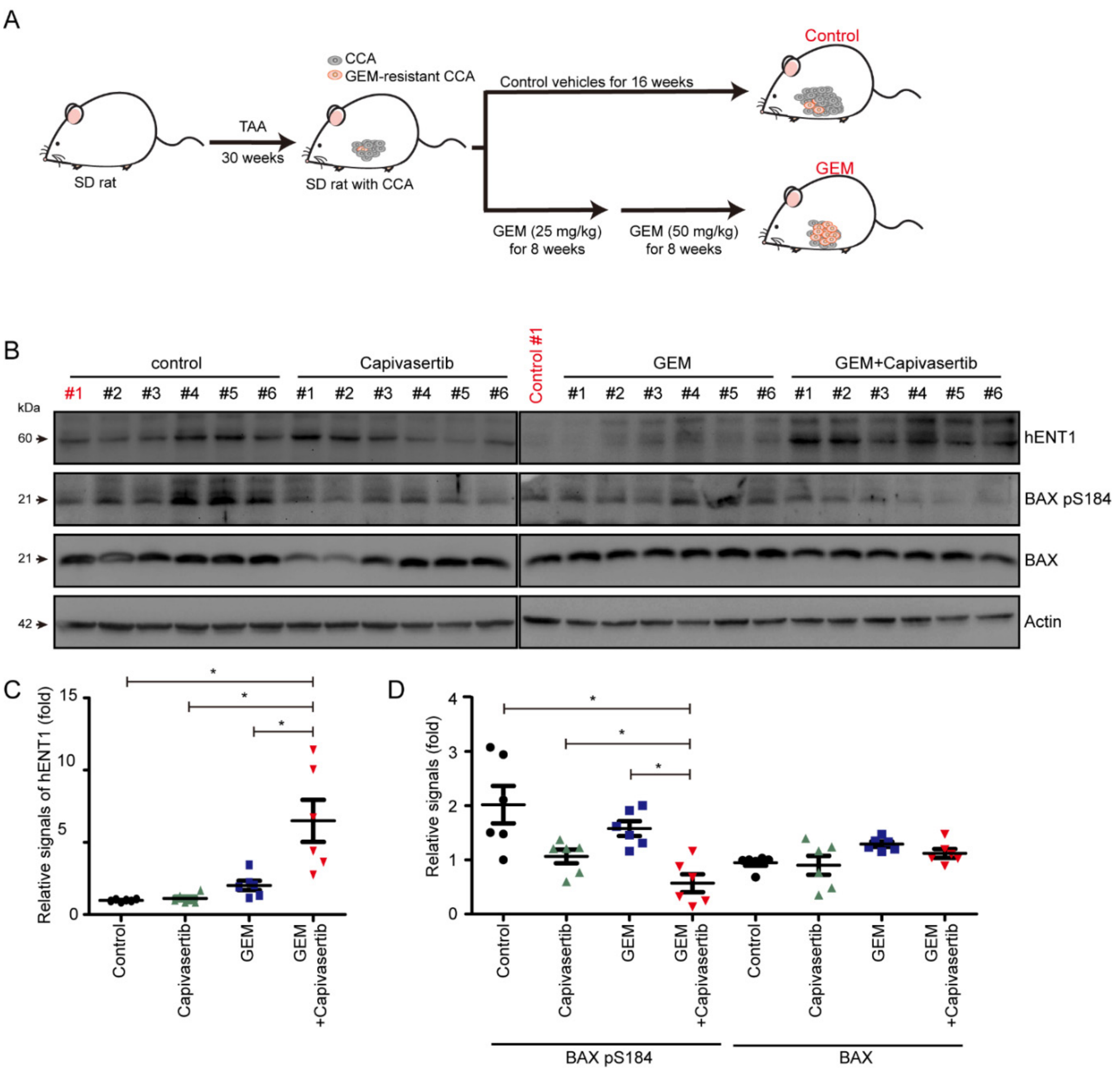

Supplementary Figure 6. hENT1 expression and BAX Ser184 phosphorylation were modulated by capivasertib in vivo.

(A) A schematic of the experimental design for the experiments presented in Figures 6A and 6B. SD rats were administered TAA for 30 weeks to induce CCA development. The rats were given 25 mg/kg GEM or control vehicle (PBS) weekly by intraperitoneal injection for 8 weeks and then 50 mg/kg GEM or control vehicle (PBS) weekly for another 8 weeks. Whole-cell lysates of the remaining rat CCA tissues were extracted, and the expression levels of the candidate proteins were analyzed.

(B) Western blots showing the protein levels of hENT1, phosphorylated BAX, and total BAX in SNU-1196-GR-derived tumor tissues in Figures 6C and 6D. Actin was used as the loading control.

(C) , (D) Quantification of relative hENT1 (C), phosphorylated BAX, and total BAX (D) signals in panel (B). Data represent the means ± SEMs. n=6 for each group. *$P < 0.05$ by Student's *t test*.

Supplementary tables

Supplementary Table 1. The characteristics of the patients with CCA

Variable	MUC4 expression		P value
	≤20	>20	
No. of patients	28	35	
Age (years)			0.954
≤65	17 (60.7%)	21 (60.0%)	
>65	11 (39.3%)	14 (40.0%)	
Sex			0.91
Male	14 (50.0%)	18 (51.4%)	
Female	14 (50.0%)	17 (48.6%)	
Performance score			0.494
0/1	23 (82.1%)	31 (88.6%)	
2	5 (17.9%)	4 (11.4%)	
Lung meta			0.132
Yes	3 (10.7%)	9 (25.7%)	
No	25 (89.3%)	26 (74.3%)	
Liver meta			0.679
Yes	9 (32.1%)	13 (37.1%)	
No	19 (67.9%)	22 (62.9%)	
Bone meta			0.684
Yes	2 (7.1%)	4 (11.4%)	
No	26 (92.9%)	31 (88.6%)	
Peritoneum meta			0.036
Yes	1 (3.6%)	8 (22.9%)	
No	27 (96.4%)	27 (77.1%)	
Distant LNs meta			0.17
Yes	6 (21.4%)	3 (8.6%)	
No	22 (78.6%)	32 (91.4%)	
Best response			0.004
PR	10 (35.7%)	2 (5.7%)	
SD	12 (42.9%)	15 (42.9%)	
PD	6 (21.4%)	18 (51.4%)	

CCA: cholangiocarcinoma

Supplementary Table 2. shRNA sequences

Gene name	Species	shRNA target sequence
LacZ		CGCGATCGTAATCACCCGAGT
MUC4 #1	*Homo sapiens*	GCCCTGATAGATTCCTGAAT
MUC4 #2	*Homo sapiens*	CGCCCTGATAGATTCCTGAAT
AKT1 #1	*Homo sapiens*	GGACAAGGACGGGCACATTAA
AKT1 #2	*Homo sapiens*	CGAGTTTGAGTACCTGAAGCT
AKT2 #1	*Homo sapiens*	TACCGCCCAGTCCATCACAAT
AKT2 #2	*Homo sapiens*	AGGACCTTCCACGTGGATTCT
AKT3 #1	*Homo sapiens*	ACTGGCAAGATGTATATGATA
AKT3 #2	*Homo sapiens*	GAAAGGGAAGAATGGACAGAA
HER2 #1	*Homo sapiens*	TGTCAGTATCCAGGCTTTGTA
HER2 #2	*Homo sapiens*	GATCACAGGTTACCTATACAT
hENT1 #1	*Homo sapiens*	CGATGCCTGGTTCATCTTCTT
hENT1 #2	*Homo sapiens*	CCTGGAATTCTACCGCTACTA
MUC4 #1	*Mus musculus*	GCCACCTCACATGACCTAATT
MUC4 #2	*Mus musculus*	GCCACCTCCTATGACCAAATT
AKT1 #1	*Mus musculus*	CCACAGTCATTGAGCGCACCT
AKT1 #2	*Mus musculus*	TCTGAGACTGACACCAGGTAT
AKT2 #1	*Mus musculus*	CGCCTCTTTGAGCTCATTCTT
AKT2 #2	*Mus musculus*	CGACCCAACACCTTTGTCATA

Supplementary Table 3. Information on the antibodies used in this study

Protein	Application (dilution)	Catalog No.	Origin	Incorporation
α-tubulin	WB (1:5000)	T6793	mouse mAb	Sigma–Aldrich (St. Louis, MO)
Actin	WB (1:5000)	MAB1501R	mouse mAb	EMD Millipore (Billerica, MA)
AKTpS473	WB (1:1000)	#4060	rabbit mAb	Cell Signaling Technology, Inc. (Danvers, MA)
pan-AKT	WB (1:2000)	#4685	rabbit mAb	Cell Signaling Technology, Inc. (Danvers, MA)
AKT1	WB (1:2000)	#2938	rabbit mAb	Cell Signaling Technology, Inc. (Danvers, MA)
AKT2	WB (1:2000)	#3063	rabbit mAb	Cell Signaling Technology, Inc. (Danvers, MA)
BAX pS184	WB (1:1000)	PA5-39778	rabbit pAb	Thermo Fisher Scientific Inc. (Waltham, MA)
BAX	WB (1:1000)	#2772	rabbit pAb	Cell Signaling Technology, Inc. (Danvers, MA)
CK-19	WB (1:2000)	ab133496	rabbit pAb	Abcam Plc. (Cambridge, UK)
ERK1/2 pT202/Y204	WB (1:2000)	#4370	rabbit mAb	Cell Signaling Technology, Inc. (Danvers, MA)
ERK1/2	WB (1:2000)	#4695	rabbit mAb	Cell Signaling Technology, Inc. (Danvers, MA)
EGFR pY1068	WB (1:1000)	#3777	rabbit mAb	Cell Signaling Technology, Inc. (Danvers, MA)
EGFR	WB (1:2000)	#4267	rabbit mAb	Cell Signaling Technology, Inc. (Danvers, MA)
HER2 pY877	WB (1:1000)	#2241	rabbit pAb	Cell Signaling Technology, Inc. (Danvers, MA)
HER2 pY1221/1222	WB (1:1000)	#2243	rabbit mAb	Cell Signaling Technology, Inc. (Danvers, MA)
HER2	WB (1:2000)	#2165	rabbit mAb	Cell Signaling Technology, Inc. (Danvers, MA)
HER3 pY1289	WB (1:1000)	#4791	rabbit mAb	Cell Signaling Technology, Inc. (Danvers, MA)
HER3	WB (1:2000)	#12708	rabbit mAb	Cell Signaling Technology, Inc. (Danvers, MA)
hENT1	WB (1:2000)	11337-1-AP	rabbit pAb	Proteintech Group, Inc. (Rosemont, USA)
Hep Par1	WB (1:1000)	MAB7927	mouse mAb	Abnova Corporation (Taipei, Taiwan)
HNF4α	WB (1:1000)	ab41898	mouse mAb	Abcam Plc. (Cambridge, UK)
MUC4	IHC (1:250)	35-4900	mouse mAb	Thermo Fisher Scientific Inc. (Waltham, MA)
MUC4	WB (1:1000)	#81692	rabbit mAb	Cell Signaling Technology, Inc. (Danvers, MA)
PARP1	WB (1:1000)	#9532	rabbit mAb	Cell Signaling Technology, Inc. (Danvers, MA)

Abbreviations: WB, western blot; IHC, immunohistochemistry; mAb: monoclonal antibody; pAb, polyclonal antibody

Supplementary Table 4. The PCR primers used in this study

Gene		Sequence (5'-3')
GAPDH	F	GTCTCCTCTGACTTCAACAGCG
	R	ACCACCCTGTTGCTGTAGCCAA
MUC1	F	AGACGTCAGCGTGAGTGATG
	R	CAGCTGCCCGTAGTTCTTTC
MUC4	F	GCCCAAGCTACAGTGTGACTCA
	R	ATGGTGCCGTTGTAATTTGTTGT
MUC16	F	AGCATCCTGGACGTAACCAC
	R	CAGGTGGAAGGGTGTTCTGT
ABCB1	F	TATGCTGGAGCAGTTCCTCA
	R	CCAGCTCCTCCTCCTTCTTT
ABCC1	F	GAAGGAAGCAAAGCAAATGG
	R	CCTGCTGATGTCCCCACTAT
ABCG2	F	CGGAAGGTGTCCTGCTACAT
	R	CTTGACCATTTCCCTTCTGC
CDA	F	AAAGCTGGCTCCTGCATAGG
	R	ACCATTTGGCTGCCTGTAGT
SLC28A3	F	ATGAATTCAGCCCTGTCCTG
	R	AAACGTGATGGCAGTTGATG
DCK	F	GATGATGTATGAGAAACCTGAACG
	R	CCAGTCTTGATAAATTGTCCACTC
SLC29A1	F	TGTGCTTCGGGCCCAAGAA
	R	TTGCCCGGAACAGGAAGGA
PRM1	F	CACATCAGAACACACATACGAC
	R	GCACTCTCAAAAGGGTATCTCA
PRM2	F	CCCGCTGTTTCTATGGCTTC
	R	CCCAGTCTGCCTTCTTCTTG
TYMS	F	CCTCTGCTGACAACCAAACG
	R	GAAGACAGCTCTTTAGCATTTG
AKT1	F	TAACCTTTCCGCTGTCGC
	R	ATGTTGTAAAAAAACGCCG
AKT2	F	GGTCGCCAACAGCCTCAA
	R	CACTTTAGCCCGTGCCTTG
AKT3	F	CTGGACATCACCAGTCCTAGCTC
	R	ACCCTTGGCTGGTCTGGG

Supplementary Table 5. Quantitative analysis of the data in Figure S2A-S2C

	SSP-25: GR/parental	SNU-1196: GR/parental	SSP-25-GR: shMUC4/shLacZ
Akt 1/2/3 S473	1.370	1.299	0.577
Akt 1/2/3 T308	0.940	1.035	0.899
AMPKα1 T183	1.296	1.230	0.810
AMPKα2 T172	1.023	0.875	0.702
Chk-2 T68	1.204	1.012	0.730
c-Jun S63	0.864	0.976	1.130
CREB S133	1.262	0.934	1.057
EGF R Y1086	1.242	1.206	0.959
eNOS S1177	3.038	0.866	1.063
ERK1/2 T202/Y204, T185/Y187	2.201	3.670	1.151
FAK Y397	1.107	0.921	0.703
Fgr Y412	1.168	0.497	-0.638
Fyn Y420	0.725	1.002	0.342
GSK-3α/β S21/S9	1.774	1.146	1.225
Hck Y411	0.918	1.244	0.273
HSP27 S78/S82	1.002	0.760	0.773
HSP60	1.397	0.940	0.882
JNK 1/2/3 T183/Y185, T221/Y223	1.108	0.968	0.837
Lck Y394	0.953	0.464	0.364
Lyn Y397	0.966	0.810	0.386
MSK1/2 S376/S360	1.097	1.167	0.898
p27 T198	1.269	0.890	1.068
p38α T180/Y182	1.263	1.212	0.810
p53 S15	0.908	0.995	1.262
p53 S392	0.734	0.928	1.075
p53 S46	0.661	0.959	0.930
p70 S6 Kinase T389	1.545	0.994	1.010
p70 S6 Kinase T421/S424	0.928	0.934	0.934
PDGF Rβ Y751	0.974	0.754	0.985
PLC-γ1 Y783	0.976	0.823	1.038
PRAS40 T246	1.604	2.189	0.795
PYK2 Y402	0.997	0.960	1.003
RSK1/2/3 S380/S386/S377	1.170	0.997	1.010
Src Y419	0.791	0.987	0.691

STAT2 Y689	0.996	0.898	0.876
STAT3 S727	1.850	1.226	1.050
STAT3 Y705	1.116	0.936	1.009
STAT5a Y694	0.880	0.577	0.445
STAT5a/b Y694/Y699	1.168	0.688	1.033
STAT5b Y699	1.112	0.547	0.634
STAT6 Y641	0.963	0.914	0.788
TOR S2448	0.937	0.922	0.687
WNK1 T60	1.056	0.852	1.037
Yes Y426	1.106	0.933	0.595
β-Catenin	0.667	0.672	0.828

Supplementary Table 6 Quantitative analysis of the data in Figure 6A

	MUC4/ GAPDH		AKT pS473/ GAPDH	
	**Control**	**GEM**	**Control**	**GEM**
#1	1.00	2.97	1.00	2.00
#2	1.03	1.66	1.35	1.28
#3	1.23	2.00	1.39	1.56
	AKT pT308/ GAPDH		AKT/ GAPDH	
	**Control**	**GEM**	**Control**	**GEM**
#1	1.00	3.81	1.00	1.01
#2	1.48	2.33	1.16	1.33
#3	1.88	2.71	0.89	1.32

Supplementary Table 7 Quantitative analysis of the data in Figure S6B

	hENT1/ Actin			
	**Control**	**Capivasertib**	**GEM**	**GEM+ capivasertib**
#1	1.00	1.29	1.33	4.39
#2	0.94	1.02	2.04	3.63
#3	1.08	0.85	2.21	2.75
#4	0.94	0.86	3.44	6.71
#5	1.05	1.68	1.16	10.05
#6	0.86	1.01	1.92	11.39
	BAX pS184/ Actin			
	**Control**	**Capivasertib**	**GEM**	**GEM+ capivasertib**
#1	1.00	1.21	1.46	1.16
#2	1.48	0.77	1.31	0.88
#3	1.51	1.23	1.16	0.68
#4	2.95	1.37	2.01	0.32
#5	3.08	1.21	1.91	0.14
#6	2.11	0.60	1.62	0.25
	BAX/ Actin			
	**Control**	**Capivasertib**	**GEM**	**GEM+ capivasertib**
#1	1.00	0.48	1.47	1.04
#2	0.68	0.35	1.24	1.19
#3	0.98	0.77	1.20	0.89
#4	0.99	1.24	1.34	1.03
#5	1.03	1.39	1.15	1.47
#6	1.00	1.17	1.35	1.10

Supplementary Table 8. Univariate and multivariate analyses of prognostic factors (OS)

Factor	Median (months)	95% C.I. of median	P value	Hazard ratio	95% C.I. of HR	P value
Age (years)			0.600	-		
≤65 (n=38)	9.69	6.76–12.62				
>65 (n=25)	7.46	4.88–10.03				
Sex			0.110	-		
Male n=32)	6.18	3.17–9.18				
Female (n=31)	9.79	7.46–12.12				
Performance score			0.112	-		
0/1 (n=54)	9.69	7.01–12.37				
2 (n=9)	6.21	3.52–8.90				
MUC4 expression			0.061			
≤20 (n=28)	11.83	9.19–14.47		Reference		
>20 (n=35)	6.87	3.63–10.10		1.01	0.57–1.79	0.969
Lung meta			0.038			
Yes (n=12)	6.18	1.77–10.58		0.75	0.36–1.58	0.447
No (n=51)	10.02	6.57–13.47		Reference		
Liver meta			0.349	-		
Yes (n=22)	4.90	2.29–7.50				
No (n=41)	10.02	7.42–12.62				
Bone meta			0.079			
Yes (n=6)	3.38	0.28–6.49		3.04	1.20–7.66	0.019
No (n=57)	9.69	6.60–12.78		Reference		
Peritoneum meta			0.054			
Yes (n=9)	7.59	0.01–16.52		2.53	1.14–5.60	0.022
No (n=54)	8.94	5.43–12.45		Reference		
Distant LN meta			0.878	-		
Yes (n=9)	9.00	6.31–11.69				
No (n=54)	8.38	5.58–11.18				
Best response						
PR (n=12)	15.77	10.14–21.40	<0.0001	Reference		
SD (n=27)	11.83	7.37–16.29		1.84	0.83–4.04	0.132
PD (n=24)	4.70	3.59–5.80		7.91	2.97–21.07	<0.0001
